# High-throughput functional profiling and evolutionary covariation analysis of entire riboswitch sequences

**DOI:** 10.1093/nar/gkag542

**Published:** 2026-06-17

**Authors:** Laura M Hertz, Anibal Arce, Elena Rivas, Julius B Lucks

**Affiliations:** Interdisciplinary Biological Sciences Graduate Program, Northwestern University, Evanston, IL 60208, United States; Department of Chemical and Biological Engineering, Northwestern University, Evanston, IL 60208, United States; Department of Chemical and Biological Engineering, Northwestern University, Evanston, IL 60208, United States; Center for Synthetic Biology, Northwestern University, Evanston, IL 60208, United States; Department of Molecular and Cellular Biology, Harvard University, Cambridge, MA 02138, United States; Interdisciplinary Biological Sciences Graduate Program, Northwestern University, Evanston, IL 60208, United States; Department of Chemical and Biological Engineering, Northwestern University, Evanston, IL 60208, United States; Center for Synthetic Biology, Northwestern University, Evanston, IL 60208, United States

## Abstract

Riboswitches are useful models for revealing how some RNA molecules undergo dynamic rearrangements of their structures to perform cellular functions. A great deal is known about riboswitch aptamer domains through sequence covariation analysis, which has been difficult to apply to expression platforms given their large sequence diversity. Here, we develop an approach to generate covariation models for entire riboswitch sequences including the aptamer domain and the expression platform. The method consists of bioinformatically extending aptamer domains to include downstream sequences and filtering these sequences using either computational or high-throughput experimental approaches to identify those that include bacterial intrinsic terminators. Filtered sequences are then used to generate covariation models. We developed this approach in the context of the fluoride riboswitch, characterizing 1901 fluoride riboswitch sequences using high-throughput *in vitro* transcription followed by next-generation sequencing, and generating a covarion model consistent with its mechanism. We then developed covariation models of the ZTP, lysine, and TPP riboswitches and find covariation support for previously published mechanisms. Our method represents a new approach to characterizing large numbers of riboswitch sequences and to generate covariation models of complete riboswitches, which should expand our understanding of riboswitch mechanisms and the evolution of RNA structure dynamics.

## Graphical Abstract

**Graphical Abstract:**
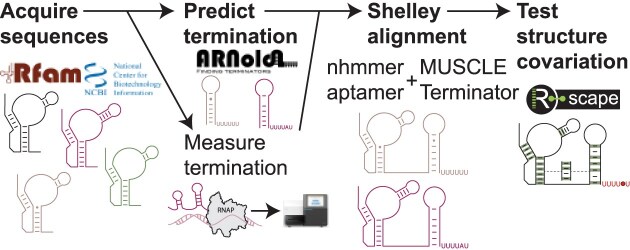

## Introduction

Riboswitches, noncoding RNA structural elements that typically reside in the 5′ untranslated region of bacterial genes, are important systems to study from fundamental principles of RNA folding [1–6] to anti-biotic targets [7–10]. Riboswitches bind a ligand in the aptamer domain, which influences the adjacent structure of the expression platform (EP) that can regulate transcription [11], translation [12], or RNA degradation [13] in bacteria, and splicing in eukaryotes [14], depending on its sequence. While much is known about how riboswitches bind their ligands [15–20], there is still much to be learned about their structural switching mechanism [21, 22].

Fundamental to aptamer discovery is sequence covariation analysis, which takes a sequence alignment as input and decodes the potential for two positions to co-vary through compensatory changes (i.e. an A and a U both changing to a G and a C) that would preserve base pairing [23–25]. The result of covariation analysis is a secondary structure model of the RNA, called a covariation “motif” that represents which base pairs are most likely given the input alignment. Aptamer domains are highly structured with the singular function of binding a ligand, providing structural conservation and thus accessible for detection through covariation analysis [20, 26–28]. In contrast, EPs, due their function, structure, and sequence diversity, have largely been missed by covariation analysis.

**Figure 1.**
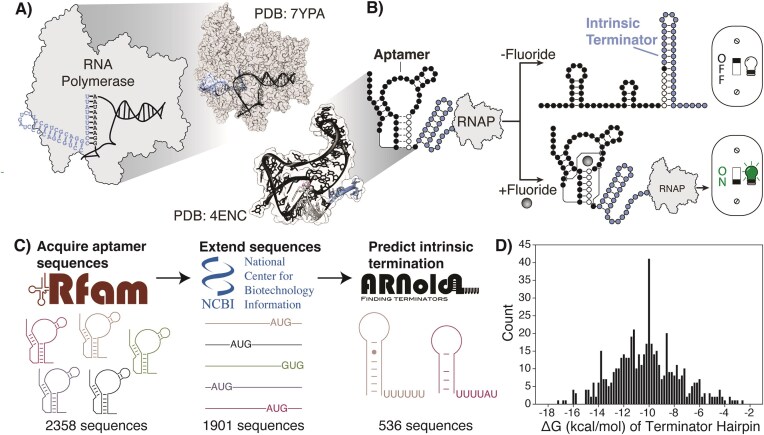
A bioinformatic pipeline to identify transcriptional fluoride riboswitches. (**A**) Left: Structure and schematic of a portion of an intrinsic terminator inside RNAP (gray) exit channel (PDB: 7YPA) with the RNA G-C rich hairpin (blue) and polyU track at the RNA:DNA (blue:black) hybrid. The terminator hairpin secondary structure is modeled from minimum free energy folding predictions from RNAStructure. Right: Structure of the fluoride aptamer (PDB: 4ENC), not to scale with the RNAP. (**B**) Schematic mechanism of a transcriptional fluoride riboswitch. In the absence of fluoride, the aptamer (black and white circle nucleotides) undergoes a structural rearrangement to form an intrinsic terminator (white and blue, and singular black, circle nucleotides) to turn off gene expression. In the presence of fluoride, the aptamer is stabilized that prevents rearrangement and allows transcription to continue. RNAP = RNA polymerase. (**C**) A bioinformatic pipeline that acquires aptamer sequences, accession ID numbers and genomic positions from Rfam family (RF01734), uses genome coordinates to extend the aptamer sequences to capture the adjacent EP sequence, and predicts which extended sequences contain an intrinsic terminator motif using the ARNold webserver. (**D**) Histogram of the ΔG (kcal/mol) of the stem–loop terminator structure predicted by ARNold.

To address this gap, we developed an approach to generate a covariation motif of a full riboswitch sequence containing both the aptamer and EPs, using high-throughput methods that filter for one specific gene regulatory function, namely intrinsic termination. Bacterial intrinsic terminators are comprised of a conserved sequence motif: a G-C rich hairpin structure followed by a poly-U track (Fig. 1A) [29, 30]. Many riboswitch ligand classes regulate at the level of transcription, and some have had their cotranscriptional folding pathways well-characterized [6, 31–33]. Notably, past work generated full riboswitch covariation models for the purine [34] and SAM [35] transcriptional riboswitches. However, the approaches used to generate these models used either structural annotation in the alignment [34] or manual curation of the alignment [35] and thus are not widely accessible to other known riboswitch classes. We therefore sought to develop a more high-throughput approach using two complementary approaches with the fluoride riboswitch (Fig. 1B) [4, 15, 36–40]. The method consists of first bioinformatically extending identified aptamer domains to include downstream sequence that could contain an EP. Sequences are then filtered using either a computational method to predict the presence of intrinsic terminators, or a high-throughput transcriptional assay that leverages massive parallel oligo synthesis (MPOS) [41] to measure function. Filtered sequences are then used to build covariation models of the entire riboswitch sequence. Using this approach, we were able to build a full covariation model of the fluoride riboswitch that expands upon the known aptamer to cover the transcription terminator, which has conserved overlap with the aptamer. This full model was similar whether the transcriptional terminators were identified computationally or experimentally, with the experimental model having fewer significant covarying base pairs. We also modified our high-throughput transcription assay to explore the functional impact of the transcription elongation factor, GreB, which rescues stalled RNA polymerases (RNAPs) and is evolutionarily conserved [42], to add to the growing body of work elucidating the role of transcription factors in riboswitch folding and function [39, 43, 44]. Finally, we used the computational filtering approach to build covariation models of the ZTP, lysine, and TPP riboswitches, to finally show conserved folding mechanisms.

## Materials and methods

### Sequence acquisition and synthesis

Fluoride riboswitch sequences were generated *in silico* by extending known aptamer sequences with downstream genomic context, and concatenating with promoter and 3′ priming site sequences according to the following workflow. First, a FASTA file of all computationally identified sequences of the fluoride aptamer were downloaded from Rfam [45] (RF01734, downloaded 8 May 2023) (see the “Data availability” section). We next calculated the length of the aptamer sequences plus the length of the *Escherichia coli* J23119 consensus promoter sequence (from the registry of standardize biological parts, https://parts.igem.org/Part:BBa_J23119), and a 3′ priming sequence used for oligo pool amplification (Supplementary Table S1). For each aptamer sequence, this total length was then subtracted from the Twist oligo pool length (300 nt), and the remaining number of nucleotides was retrieved from the genome entry for that aptamer sequence from NCBI database via Entrez (Entrez Programming Utilities Help, available at https://www.ncbi.nlm.nih.gov/books/NBK25501/), starting from the 3′ end of the aptamer sequence. The *E. coli* J23119 consensus promoter sequence was then prepended to each aptamer extended sequence, and the 3′ oligo priming site was appended to each sequence. We note that we removed the first five nucleotides from the Rfam sequence entry of the genomically encoded region as we observed that the Rfam database included the 10 nucleotides leading up to the fluoride aptamer (starting at P1) and leader sequences can impact riboswitch function [31, 32]. The final sequences were exported into a spreadsheet.

Entrez was used again to collect information about what protein the aptamer was potentially regulating and the location of the start codon. The information was added to the sequence information used for the Twist Bioscience sequence pool order (Supplemental Document A, “Bioinformatics” sheet).

Code was executed through a Jupyter Notebook running python (v. 3.10.9). The prepping_sequences.py script was used to generate the fluoride riboswitch sequences and oligo pool order. The NCBI_CDS.py script was used to identify the potential protein.

### Computationally predicting intrinsic termination

We next classified extended fluoride aptamers by whether they are predicted to undergo intrinsic termination using the online webserver, ARNold [46]. To do this, extended sequences were converted into a FASTA file format. The FASTA file was then submitted to the ARNold webserver [46]. The results were then processed to create a FASTA file of fluoride riboswitches post-aptamer sequences between 90 and 110 nts in length that are predicted to undergo intrinsic termination.

Code was executed through the script CDS_ARNold.py on a Jupyter Notebook running python (v. 3.10.9).

### Covariation analysis for computationally predicted fluoride riboswitches

Covariation analysis on fluoride riboswitch terminating variants (see the “Computationally predicting intrinsic termination” section) was done through analyzing a multiple sequence alignment with the R-scape (RNA Structural Covariation Above Phylogenetic Expectation) package [24] (v2.0.0.j). A multiple sequence alignment was first created for the aptamer region, another for the post-aptamer sequences, and then both alignments were merged for R-scape analysis.

To create the alignment for the aptamer region, the fluoride aptamer Stockholm alignment file was downloaded from Rfam (RF01734.sto). From the ARNold FASTA output of CDS_ARNold.py, the aptamer region was aligned to RF01734.sto using nhmmer [47], which created the output Predicted_RF01734_profile.sto.

To create the alignment for the extended post-aptamer sequences, we took the output from CDS_ARNold.py and Predicted_RF01734_profile.sto to generate a FASTA file of the post-aptamer sequences. The post-aptamer sequences were aligned using Jalview (v. 2.11.3.3) and the accessory MUSCLE with default parameters [48].

The two separate alignments were then merged using the Shelley_Alignment.py script through a Jupyter Notebook running python (v. 3. 10.9). R-scape was used to assess significant covariation on the compiled alignment, and to build a consensus secondary structure informed by covariation using the CaCoFold algorithm [24].

Code was executed through a Jupyter Notebook running python (v. 3.10.9). The Shelley_Alignment.py script was used to assess aptamer alignment, generate the FASTA file for MUSCLE alignment, and merge the aptamer and post-aptamer alignments.

### Covariation analysis for experimentally validated transcriptional fluoride riboswitches

Covariation analysis on fluoride riboswitch variants that were validated to undergo intrinsic termination through next-generation sequencing (NGS; see the “Read analysis” section) were saved in a FASTA file (see the “Data availability” section). We then performed a similar alignment and R-scape analysis as in the “Covariation analysis for computationally predicted fluoride riboswitches” section with the exception that the post-fluoride aptamer sequence lengths were limited between 5 and 40 nts to generate the MUSCLE alignment.

The Stockholm file output from running CaCoFold was used to generate a covariation model to align all terminators validated through NGS. We used the Infernal package to build a covariation model with cmbuild, calibrated the model with cmcalibrate, and searched all validated terminators with cmsearch. We then generated the covariation model of all validated terminators by running CaCoFold from R-scape.

### Covariation analysis for additional riboswitch classes

We performed similar riboswitch covariation analysis for the purine (RFAM RF00167), SAM (RFAM RF00162), ZTP (RFAM RF01750), lysine (RFAM RF00168), TPP (RFAM RF00059), and glmS (RFAM RF00083) aptamer families. Sequences were extended following the “Sequence acquisition and synthesis” section, classified as containing an intrinsic terminator following the “Computationally predicting intrinsic termination” section, and then subject to analysis following the “Covariation analysis for computationally predicted fluoride riboswitches” section. During this process, the post-aptamer sequences were limited by length (post-purine aptamer sequences were limited to 45–65 nucleotides, post-SAM aptamer sequences to 70–80 nucleotides, post-ZTP aptamer sequences to 5–35 nucleotides, post-lysine aptamer sequences to 25–50, post-TPP aptamer sequences to 40–45 nucleotides, and post-glmS aptamer sequences to 30–100 nucleotides) to produce a clean alignment. The first pass analysis was restricted to the first 1000 sequences due to limits on the MUSCLE alignment webserver. Once CaCoFold was run on the combined alignment, a new covariation model was calibrated and searched through all extended sequences following the “Covariation analysis for experimentally validated transcriptional fluoride riboswitches” section, except that the models were searched through all downloaded Rfam sequences to expand the motif analysis to all potential EPs.

### Oligo pool library amplification

The single-stranded DNA (ssDNA) oligo pool from Twist (see the “Sequence acquisition and synthesis” section) was resuspended to 20 ng/µl. A 50 µl polymerase chain reaction (PCR) was set up to amplify 20 ng of DNA: 1 µl of ssDNA pool (20 ng/µl), dNTPS (NEB, N0447L) to final concentration of 200 µM each, Q5 Reaction Buffer (NEB, B9027S) to a final concentration of 1×, primer A and primer B (Supplementary Table S2) to a final concentration of 300 nM each, 0.5 µl of Q5 polymerase (NEB, M0491L), and water to a final volume of 50 µl. In a thermocycler (BIO RAD, S1000TM), the reaction was heated to 95°C for 3 min and then cycled 15 times through 98°C for 20 s, 53°C for 15 s, and 72°C for 15 s. The reaction was held at 72°C for an additional 2 min to complete polymerase extension. The double-stranded DNA (dsDNA) product was purified using Sera-Mag Select Beads at a ratio of 1.5× (cytiva, 29343052), following the manufacturer protocol. This consisted of vortexing beads with the 50 µl PCR reaction, incubating at room temperature for 5 min, placing on a magnetic stand, removing supernatant, washing beads twice with freshly prepared 85% ethanol, air drying beads, and eluting dsDNA with 20 µl of water. The dsDNA concentration was quantified using a High Sensitivity Qubit assay (Invitrogen, Q32854). From this PCR reaction, 100 ng of dsDNA went through a second round of amplification following the same protocol to produce enough dsDNA for *in vitro* transcription (IVT). Input library quality was assessed by electrophoresis using the DNA 1000 assay on the Bioanalyzer (Agilent, 2100 bioanalyzer).

We conducted a PCR to prepare the oligo pool for NGS to assess the sequence representation within the oligo pool. The PCR reaction was downscaled to 25 µl reaction: 0.5 µl of ssDNA pool (20 ng/µl), 0.75 µl of 10 mM dNTPs (deoxynucleotide), 5 µl of 5× Q5 Reaction Buffer, 0.75 µl of 10 µM of each primer C and primer D (Supplementary Table S2), 0.25 µl of Q5 polymerase, and water to a final volume of 25 µl. The reaction was then thermocycled as follows: 95°C for 3 min, then cycled 2 times through 98°C for 20 s, 54°C for 15 s, 72°C for 15 s, then cycled 12 times through 98°C for 20 s, 65°C for 15 s, 72°C for 15 s, and then 72°C for 1 min, then cooled to 12°C. The reaction was purified using the using Cytiva Sera-Mag Select Beads at a ratio of 1.0×. Notably, the sequencing was performed with a 2 × 150 cycle kit (Supplemental Document A, “Pool validation” sheet).

### GreB protein purification

The GreB transcription elongation factor was purified following previously published methods with minor modifications [49]. The expression plasmid was generously provided by Irina Artsimovich. Briefly, *E. coli* BL21 (DE3) cells were transformed with the pIA577 plasmid encoding GreB with a C-terminal 6× His tag [49]. A single colony was grown in 1 l of auto-induction terrific broth media (Boca scientific, GCM19.0500) supplemented with kanamycin (50 µg/l). Cultures were incubated at 37°C with shaking at 240 rpm until the OD_600_ reached ~0.6, after which the temperature was reduced to 20°C for overnight protein expression. The next morning, cells were harvested by centrifugation at 5000 × *g* for 10 min at 4°C, and the pellets were resuspended in 40 ml of lysis buffer containing 1 M NaCl, 40 mM Tris (pH 7.5), 5 mM TCEP (tris(2-carboxyethyl)phosphine), 5% glycerol.

Cell lysis was performed via sonication on ice using a 12-mm tip placed inside the 50 ml falcon tube with the cell pellet and conditions set at 50% amplitude, pulse cycle: 10 s ON and 20 s OFF for 5 cycles in total, and the lysate was clarified by centrifugation at 15 000 × *g* for 30 min. The supernatant was filtered through a 0.45-µm syringe filter and incubated with 5 ml of Ni-NTA agarose beads (Goldbio H-350-25) that were previously washed and pre-equilibrated with lysis buffer, for 1 h at 4°C with rotation mixing. The resin was loaded into a gravity column and washed with 50 ml of wash buffers containing each 20 mM and 50 mM imidazole to remove nonspecifically bound proteins. The target proteins were eluted with 500 mM imidazole and fractions of 5 ml were collected.

Elution fractions containing the target protein with minimal impurities were identified by sodium dodecyl sulfate–polyacrylamide gel electrophoresis (SDS–PAGE), pooled, and dialyzed overnight at 4°C against dialysis buffer (2 M NaCl, 80 mM Tris, pH 7.5, 2 mM TCEP, 5% glycerol). The protein was subsequently concentrated and stored at −20°C in storage buffer (1 M NaCl, 80 mM Tris, pH 7.5, 2 mM TCEP, 50% glycerol). Protein identity and mass were confirmed by mass spectrometry. Importantly, the His tag was not removed in this protocol.

### Single- and multiple-round *in vitro* transcription with fluorescent output

To evaluate the effect of GreB in single- and multiple-round (runoff) IVT reactions, we adapted a standard transcription protocol to enable real-time fluorescence measurements following open complex formation. Reactions were prepared in conditions with or without 1.2 µM GreB and with or without 10 µg/ml rifampicin [50] (Sigma–Aldrich, R3501-250MG) to distinguish single- and multiple-round transcription, respectively. Briefly, the DNA template used for IVT consisted of the constitutive *E. coli* pJ23119 consensus promoter (from the registry of standardize biological parts) driving the expression of the 3WJdB fluorogenic RNA aptamer [51]. The 3WJdB template was generated using PCR with primers E and F (Supplementary Table S2), followed by a PCR cleanup (Qiagen, 28506) and concentrated to 1.3 µM stock. The dye DFHBI-1T (Tocris, 5610) was dissolved in dimethyl sulfoxide (DMSO) to 40 mM. To allow open complex formation, 2.5 pmol of template DNA was incubated with 2 units of *E. coli* RNAP (NEB, M0551S) in 20 µl of buffer containing 62 mM KCl, 1.25 mM dithiothreitol (DTT), 25 mM Tris (pH 8), 6.25 mM MgCl_2_, 0.125 mM ethylenediaminetetraacetic acid (EDTA), 0.21 mM DFHBI-1T, and 25 ng of bovine serum albumin (BSA). Where indicated, 2 µl of 15 µM purified GreB was added to the reaction or water for noGreb. The reaction was then incubated at 37°C for 10 min to allow open complex formation. Transcription was initiated by adding 2.5 µl of a start solution containing 5 mM NTPs. For single-round reactions, this start solution also contained 100 µg/ml rifampicin, resulting in a final rifampicin concentration of 10 µg/ml in the reaction to inhibit reinitiation (single-round start solution). In multiple-round reactions, rifampicin was omitted and substituted with water. Two technical replicates of 5 µl from each reaction were transferred to a well of a 384 well plate (Corning, 3544) sealed with optically clear adhesive film (Thermo, 232701) and incubated at 37°C in a plate reader (BioTek Synergy H1, H1M). Fluorescence intensity (Ex: 485 nm; Em: 520 nm; gain = 60) was measured every 3 min for 7 h. Each experiment was performed in triplicate. Data are in Supplemental Document B.

To convert fluorescence arbitrary units to µM FITC equivalents, a standard calibration curve was generated using a NIST-traceable FITC standard solution (Thermo Fisher, F36915), yielding a linear fit with *R*^2^ = 0.9999 and a slope of 10 446. This calibration was used to convert raw fluorescence data to µM FITC equivalents (Supplementary Fig. S1 and  Supplemental Document B).

## RNA-Seq for next-generation sequencing

**Figure 2.**
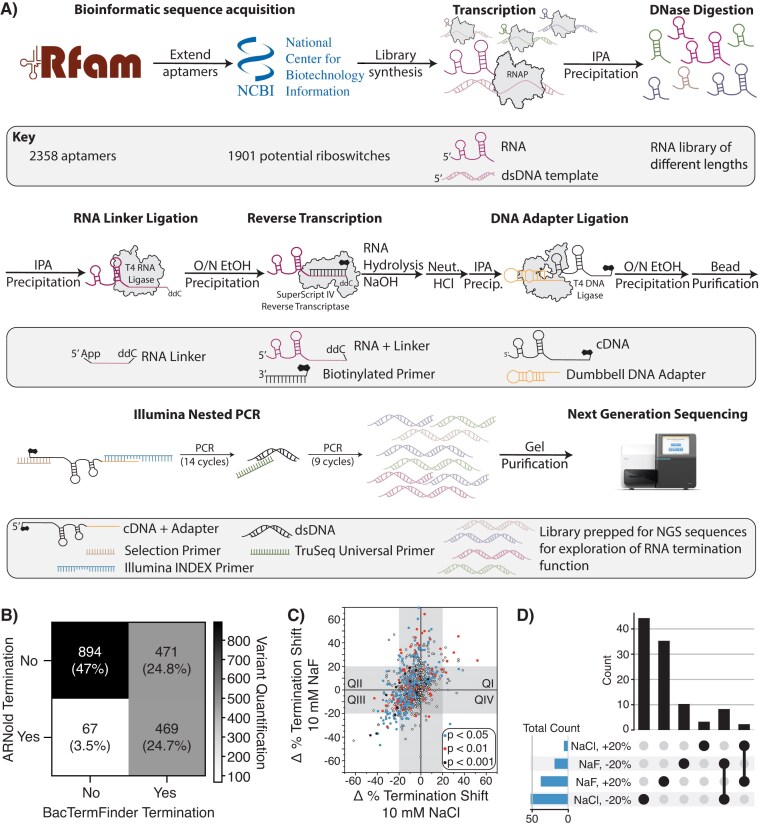
Experimental pipeline for high-throughput assessment of transcriptional riboswitch function. (**A**) Molecular schematic from acquiring aptamer sequences from Rfam to generating an NGS library to characterize transcriptional read through. 2358 Fluoride aptamer (RF01734) sequences were collected from RFam and extended through NCBI genome records, resulting in 1901 riboswitch sequences. Promoter and priming site sequences were appended to create DNA templates, which were then synthesized and amplified. The DNA library was transcribed with the *E. coli* RNAP (PDB: 7YPA) in the presence of 10 mM NaCl or NaF and either 0 or 1.2 µM GreB. Cleanup was performed to isolate RNA constructs from proteins and DNA. To prepare the RNA library for NGS, the 3′ end was ligated (PDB: 1S68) with a known RNA sequence (light pink, Linker) to serve as the reverse transcriptase (PDB: 1MML) priming site. Then the 5′ end of the resulting cDNA (black) was ligated (PDB: 6DT1) with a known DNA sequence (orange, Dumbell) and amplified using Illumina Primer sequences. The prepared DNA sequencing library underwent NGS to identify reads as either terminated or anti-terminated, which were then counted for each variant and in each condition. (**B**) Confusion matrix tabulating which riboswitch sequences were predicted to be transcriptional regulators by ARNold versus those measured to be transcriptional regulators through the NGS assay without GreB and analyzed by BacTermFinder. The count and percent of variants for each category is written. The shading is for quantity comparison between the different conditions. (**C**) The difference of percent termination reads (1.2–0 µM GreB) for each variant in 10 mM NaCl (*x*-axis) and 10 mM NaF (*y*-axis). Variants in QI represent those that have increased OFF and ON signals in the presence of GreB, QII represent those that have reduced OFF and increased ON signals and thus higher dynamic range in the presence of GreB, QIII represent those that have reduced OFF and ON signals in the presence of GreB, QIV represent those that have increased OFF and decreased ON signals and thus lower dynamic range in the presence of GreB. A gray box encompasses −20%:20% for each salt condition to highlight the variants outside the box that have a change in termination by 20%. The Student’s *t*-test was done for all comparisons with statistically significant variant changes being colored blue (*P* < .05), red (*P* < .01), and black (*P* < .001). (**D**) An UpSet plot to visualize the intersect of variants in the various conditions with possible outcomes of >|20%| change in termination rate with GreB that are statistically significant (*P* < .05) for NaCl or NaF. Total number of variants for each condition to the bar graph on the left (blue bars).

The following steps were used for sequencing-based assay: single-round IVT of oligo pool, DNase digestion, 3′ linker ligation, reverse transcription (RT), 3′ complementary DNA (cDNA) adapter ligation, sequencing library preparation, and gel purification (see Fig. 2A).

### Single-round *in vitro* transcription of oligo pool (high throughput)

A 25 µl IVT reaction was prepared from the following: 2 µl of 1 M Tris (pH 8.0), 0.02 µl of 0.5 M EDTA (pH 8.0) (Invitrogen, 15575020), 1 µl of 0.1 M DTT (single-use frozen aliquot), 2.5 µl of 50 mM MgCl_2_ (NEB, B0510A), 0.25 µl of BSA (NEB, B9000S), 4.2 µl of 116 ng/µl amplified dsDNA template pool (final concentration of 100 nM), 2 µl of *E. coli* Holoenzyme (NEB, M0551S), either 2.5 µl of 100 mM of NaCl or NaF, and water (Invitrogen, 10977-015) up to 20.5 µl. Another 2 µl came from either water or GreB, which was first diluted in water to 15 µM and then added to the sample for a final transcription reaction concentration of 1.2 µM. The samples were incubated at 37°C for 10 min. The transcription reaction was started through addition of 2.5 µl of single-round start solution that included 5 mM NTPs for a final concentration of 500 µM (125 µM of each nucleotide) and rifampicin [50] (Sigma–Aldrich, R3501-250MG) to a final concentration of 10 µg/ml. Transcription was conducted for 5 min at 37°C. The reaction was quenched by adding 75 µl of TRIzol (ambion, 15596018), followed by 20 µl of chloroform (Acros organics, 423555000). The reactions were briefly vortexed and incubated at room temperature for 2 min. They were then centrifuged for 5 min at 4°C at 12 000 *g*. The top 70 µl of the aqueous layer was placed into a new tube, followed by the addition 7 µl of 1 M NaCl, 1 µl of GlycoBlue (Invitrogen, ref. AM9515), and 50 µl isopropanol (Sigma–aldrich, 190764-1L). The reactions were inverted several times, incubated at room temperature for 10 min, and centrifuged for 10 min at 4°C at 21 130 *g*. The samples were then washed with 400 µl of 70% ethanol, centrifuged for 1 min at 4°C at 21 130 *g*, and air-dried for 5 min.

### DNase digestion

Nucleic acid samples from single-round transcription of the oligo pool were resuspended in 50 µl containing: 43 µl of RNase-free water, 5 µl of 10× TURBO DNase buffer (Invitrogen, 4022G), and 2 µl of TURBO DNase (Invitrogen, AM2238), and incubated at 37°C in a thermocycler for 60 min. The reaction was then quenched with 150 µl of TRIzol and 40 µl of chloroform, incubated at room temperature for 2 min, and centrifuged at 4°C for 5 min. The top 140 µl of aqueous phase was extracted into a new tube. Into this was added 14 µl of 1 M NaCl, 1 µl of GlycoBlue, and 100 µl of isopropanol, followed by inversion several times and incubation at room temperature for 10 min. This mixture was then centrifuged for 10 min at 4°C at 21 130 *g*. The samples were washed with 400 µl of 70% ethanol, centrifuged for 1 min at 4°C at 21 130 *g*, and air-dried for 5 min. The RNA samples were resuspended in 9 µl of RNase-free water.

### 3′ Linker adenylation

A 3′ linker was then ligated to the post-DNase digested transcription products with the following procedure, which first adenylated the 5′ end of the linker. 5′ linker adenylation was performed with a reaction containing: 1.25 µl of 100 µM of oligo G (Supplementary Table S2), 2.5 µl 1 mM ATP (NEB, N0757A), 2.5 µl 10× NEB 5′ Adenylation Buffer (NEB, M2610S), and water to 22.5 µl. The reaction was scaled up to 100 µl and 22.5 µl was aliquoted into 0.2 ml PCR tubes. To each reaction, 2.5 µl of Mth RNA ligase (NEB, M2611AA) was added. Reactions were incubated at 65°C for 1 h. The reactions were combined and 300 µl of TRIzol was then added to the pooled reactions, which were then incubated at room temperature for 5 min. Eighty microliters of chloroform was then added to the reactions, followed by incubation at room temperature for 2 min. The samples were then centrifuged at 21 130 *g* for 15 min at 4°C. The top 280 µl of the aqueous phase was extracted into a new tube. Into this was added 1 µl of GlycoBlue, and 200 µl of isopropanol, followed by inversion several times and incubation at room temperature for 10 min. This mixture was then centrifuged for 10 min at 4°C at 21 130 *g*. The samples were washed with 400 µl of 70% ethanol, centrifuged for 1 min at 4°C at 21 130 *g*, and air-dried for 5 min. The RNA linker pellet was dissolved in 20 µl of nuclease-free water and quantified by Qubit. The RNA molarity (MM: 6782.1 g/mol) was used to determine the final concentration for the linker ligation reaction.

### 3′ Linker ligation

The linker ligation was performed with a reaction containing: 6 µl of 50% PEG 8000 (NEB 1004S), 2 µl of 10× T4 RNA Ligase Buffer (NEB B0216S), and 2 µl of 200 nM 5′ App Linker prepared as above. The reaction mixture was vortex followed by the addition of 0.5 µl of SuperaseIn RNAse Inhibitor (Invitrogen, AM2696), 0.5 µl of T4 RNA ligase, and 0.5 µl of Trunc KQ (NEB, M0373L) was added and mixed by pipetting up and down 10 times. A master mix according to the number of samples was made, and then 11 µl of the master mix was distributed to each 9 µl sample. The final 20 µl reaction was incubated at 25°C in a thermocycler for 2 h. Reactions were then ethanol precipitated by adding 130 µl of water, 15 µl of 3 M NaOAc (Invitrogen, AM9740), 1 µl of GlycoBlue, and 450 µl of ethanol. The samples were inverted several times and incubated at −20°C overnight. The samples were then centrifuged for 30 min at 21 130 *g*, washed with 70% ethanol, centrifuged for 1 min at 4°C at 21 130 *g*, and air-dried for 5 min. The linker-ligated RNA samples were then resuspended in 3 µl of RNase-free water.

### Reverse transcription

To perform RT, 3 µl of 0.1 µM RT primer H (Supplementary Table S2) was added to each linker-ligated sample and then incubated at 95°C for 2 min, 65°C for 5 min, and held at 25°C to anneal the RT primer. During this time, an RT enzyme reaction was prepared containing: 7.5 µl water, 4 µl of 5× SSIV Buffer (Invitrogen, 18090010), 1 µl of 10 mM dNTPs (cNEB, N0447L), 1 µl of 0.1 M DTT (single-use frozen aliquot, Invitrogen, 18090010), and 0.5 µl SSIV (Invitrogen, 18090010). A master mix was made according to the number of samples. After the samples had been at 25°C for at least 1 min, 14 µl of the master mix was added and mixed by pipetting. The complete reaction was incubated at 25°C for 2 min, 50°C for 10 min, 80°C for 10 min, and held at 4°C. After the reaction had been at 4°C for at least 1 min, 1 µl of 4 M NaOH was added to hydrolyze the RNA, mixed by pipetting, briefly vortexed and spun down. The reactions were then heated at 95°C for 5 min and then cooled to 4°C. Two microliters of 1 M HCl was added to each reaction to neutralize pH, followed by 17 µl of isopropanol. The samples were then incubated at room temperature for 10 min, centrifuged at 4°C for 10 min at 21 130 *g*, washed with 70% ethanol, centrifuged for 1 min at 4°C at 21 130 *g*, and air-dried for 5 min. The resulting cDNA samples were resuspended in 7 µl of RNase-free water.

### 3′ cDNA adapter ligation

Each cDNA reaction was then ligated on the 3′ end to a Dumbbell adapter following the StructureSeq 2.0 protocol [52]. Into each cDNA reaction was added 0.5 µl of 100 µM of SS2.0 Dumbbell (Oligo I; Supplementary Table S2). Samples were incubated on a thermocycler at 95°C for 2 min and then cooled to 21°C for 3 min. During this time, a ligation mix was prepared containing: 2.5 µl 10× T4 DNA Ligase Buffer (NEB B0202S), 2.5 µl of 5 M Betaine (Sigma, B0300-1Vl), 10 µl of 50% PEG8000 (NEB, B1004S), and 2.5 µl T4 DNA Ligase (NEB, B0202S). The reaction mix was scaled up according to the number of samples and then aliquoted to each cDNA sample. Reactions were then incubated at 30°C for 2 h, at 65°C for 15 min to deactivate the enzyme, and cooled to 4°C. Into each reaction, 125 µl of RNase-free water, 15 µl of 3 M NaOAc, 1 µl GlycoBlue, and 450 µl of cold ethanol were added. The samples were left at −20°C overnight. Samples were spun down for 30 min at 21 130 *g* for 30 min at 4°C, washed with 70% ethanol, centrifuged for 1 min at 4°C at 21 130 *g*, and air-dried for 5 min. The ligated cDNA was then resuspended in 50 µl of nuclease free water. To remove excess Dumbbell and RT primer, bead purification was done with Agencourt AmpureXP (Beckman Coulter, A63881) at 1.8× ratio according to the manufacture’s protocol. The samples were resuspended in 20 µl of nuclease-free water and stored in −20°C.

### Sequencing library preparation

Sequencing libraries were prepared through PCR reactions on the ligated cDNA to complete adapter sequences and add necessary indexes. PCR reactions were assembled by first combining 5 µl of each ligated cDNA sample, 0.25 µl of 100 µM Index primer (Oligo J; Supplementary Tables S2 and  S3) in a PCR tube. To add sequence variability for the start of the sequencing reads, two different selection primers were used, primer K for NaCl conditions and primer L for NaF conditions (Supplementary Table S2). For each selection primer a master mix was made consisting of 21 µl of nuclease free water, 10 µl of 5× Q5 Rxn buffer, 12.5 µl of 100 nM Primer K or L, 0.5 µl of 10 mM dNTPs, and 0.5 µl Q5 polymerase. The master mix was aliquoted in 44.5 µl to the respective tube, i.e. primer K to NaCl conditions and primer L to NaF conditions. The following thermocycler protocol was then run: 98°C for 30 s, then cycled 14 times through 98°C for 10 s, 65°C for 30 s, 72°C for 30 s, 12°C forever. At this point, 0.25 µl of 100 µM Illumina TruSeq Universal Adapter (Oligo M; Supplementary Table S2) was added to each reaction. The PCR reaction continued with 9 cycles through 98°C for 10 s, 65°C for 30 s, 72°C for 30 s, and then 72°C for 5 min and 4°C for 3 min. At this point, 0.25 µl of ExoI (NEB, M0293S) was added to each reaction to remove excess primer. The reactions were then incubated at 37°C for 30 min, followed by a 20 min enzyme deactivation step at 80°C, and cooling to 4°C. The samples were then transferred to a 1.7-ml tube for PCR clean up using 5× Buffer PB (Qiagen, 19066), applied to a silica spin column (NEB, T1017L), vacuumed, washed twice with 750 µl of Buffer PE (Qiagen, 19065), vacuumed, placed back into a collection column, and dry spun for 1 min at 21 130 *g*. The reaction was eluted in 15 µl of water and spun for 1 min at 21 130 *g*.

### Gel purification of sequencing library

Sequencing libraries were loaded into an 8% denaturing gel (national diagnostics, EC-830, EC-835, EC-840) and run at 18 W for 2 h. The gel was stained with 3 µl of SybrGold (Invitrogen, S11494) for 5 min in 0.5× Tris-Borate-EDTA (TBE) buffer (RPI, T60040-1000; Sigma, B7901; Invitrogen, 15575020) while rolling and visualized with the SmartBlueTM Transilluminator (Accuris, E4000). Each sequencing library (between 200 and 404 bp) was then cut out and eluted overnight in 1 ml of 1× buffer TE (IDT, 11-05-01-13) rotating at 4°C in a 2-ml tube. Due to tube size limitation, the 1 ml elution reactions were split into 500 µl into 1.7 ml tubes for ethanol precipitation. To each tube 60 µl of 3 M NaOAc, 1 µl GlycoBlue, and 1250 µl of cold ethanol was added. Reactions were incubated at −70°C for 30 min and centrifuged at 21 130 *g* for 30 min at 4°C, washed in 70% ethanol, centrifuged for 1 min at 4°C at 21 130 *g*, and air-dried for 5 min. The sequencing libraries were resuspended in 11 µl of nuclease-free water and concentrations measured using the High Sensitivity Qubit assay (Invitrogen, Q32854).

### Next-generation sequencing

To assess the success of the gel purification prior to NGS, the purified libraries were run on an 8% denaturing gel for 2 h at 18 W (Supplementary Fig. S2A). The libraries were sent to NUSeq Core Facility for Paired End, 150-cycle sequencing on the Element AVITI (System: 880-00001). The core performed additional quality control with a 2100 bioanalyzer (Supplementary Fig. S2B) and KAPA qPCR quantification prior to loading.

### Read analysis

FASTQ Paired End files were combined using PEAR (v0.9.6) [53] using the following command line: pear -f [library_#_R1].fastq.gz -r [library_#_R2].fastq.gz -o library_#/ library_#_PEAR -y 4G -j 1, where the # symbol refers to the sample number (Supplemental Document A). The reads were aligned to their accession number and genome positions using STAR (v2.7.9a) [54]. A synthetic genome was made from the pool of sequences (Supplemental Document A, “Bioinformatics” sheet) by removing the J23119 promoter sequence and formatting the sequences into a FASTA format with their accession IDs. The FASTA file (see the “Data availability” section) was turned into a genome assembly using megahit (v1.0.6.1) and calibrated with the genomeGenerate mode from STAR.

To align the reads to the reference genome, STAR was run with the following specified parameters: --alignIntronMax 1 --alignEndsType Local --alignSoftClipAtReferenceEnds Yes --outFilterScoreMinOverLread 0.5 --outFilterMatchNminOverLread 0.5 --outSAMmultNmax 1 (Supplementary Fig. S3). The generated output SAM file was used to quantify reads through the Read_Classification_BacTermFinder.py script on a Jupyter Notebook running python (v. 3.10.9). Reads were filtered out if the start position of the alignment was not within the beginning of the riboswitch sequence. Termination sites were identified with BacTermFinder with the following command: python genome_scan.py Variant_Genome.fasta 3 out 10 000 as suggested [55]. The top scoring termination window above the recommended threshold of 0.47 was used to classify reads as “terminated.” Results from this analysis are within the file Supplemental Document A, “Rep1_output” and “Rep2_output” sheets. The Pearson correlation coefficient (*r*) was used to assess the read count similarity between technical replicates (Supplementary Table S5) in Excel using the command: =PEARSON([array1],[array2]).

Data plotting was done with DataGraph (v4.5.1) and python 3 (v. 3. 10.9, confusion_matrix, ConfusionMatrixDisplay, matplotlib).

### Calculation of percent anti-termination

Percent anti-termination (%AT) was calculated by the formula 1-“terminated” reads/total reads (Supplemental Document A and Supplementary Fig. S4). The %AT were averaged between the two replicates and the change (Δ) of %AT between conditions was calculated (%AT NaF − %AT NaCl). Then the reads were classified as “transcriptional” or “translational,” where variants were marked as “Translational” if their %AT was greater than 95%. If “transcriptional,” the variants are further categorized into “Off” if Δ%AT < −20%, “non-functional” if −20% < Δ%AT < 20%, “low” if 20% < Δ%AT < 40%, “mid” if 40% < Δ%AT < 50%, and “high” if 50% < Δ%AT. Then, the Δ%AT change between 0 and 1.2 µM of GreB was assessed. If GreB altered the Δ%AT by at least 20%, then GreB was noted as impacting riboswitch function.

### Single dsDNA template preparation for individual *in vitro* transcription

Individual riboswitch sequences were ordered as gBlocks (IDT) that matched their sequence in the oligo pool and resuspended to 10 ng/µl. A 50 µl PCR mix was made of: 5 µl 10× Taq Buffer (NEB, B9014S), 1 µl 10 mM dNTP mix (NEB, N0447L), 0.25 µl of 100 µM Primer A (Supplementary Table S2), 0.25 µl of 100 µM Primer B (Supplementary Table S2), 0.25 µl of 10 ng/µl gBlock, and 0.25 µl Taq DNA Polymerase (NEB, M0273X), and water to 50 µl. The reaction was then heated to 95°C for 3 min, then cycled 25 times through 95°C for 45 s, 53°C for 30 s, and 68°C for 1 min. A final 68°C extension occurred for 5 min, and then the reaction was cooled to 12°C. The PCR reaction was cleaned up by adding 5× Buffer PB (Qiagen, 19066) and applying the solution to a spin column (Epoch life science, 1910-250). The sample was washed twice with 750 µl of Buffer PE (Qiagen, 19065), spun for 1 min to remove excess liquid, and eluted in 40 µl of RNase-free water (Invitrogen, 10977-015). DNA template concentrations were measured using the Broad Range Qubit assay (Invitrogen, Q32853).

### Single-round *in vitro* transcription of single riboswitches (low throughput)

To validate the NGS results, the single-round IVT with GreB was repeated with dsDNA templates of individual sequences (Supplemental Document A, “Low-throughput validation” sheet). After DNase digestion, the samples were resuspended in 10 µl of RNase-free water. Ten microliters of 2× loading buffer (8 M urea with bromophenol blue dye; Sigma–Aldrich, B0126-25G) were added to the samples and the samples were run on a 10% denaturing gel (national diagnostics, EC-830, EC-835, EC-840) at 18 W for 90 min with 0.5 µl of 1% ssRNA Century Plus Ladder (Invitrogen, AM7145). The gel was stained for 5 min with 3 µl of SybrGold gel (Invitrogen, S11494) in 40 ml 0.5× TBE gel (RPI, T60040-1000; Sigma, B7901; Invitrogen, 15575020) buffer while rolling. The gel was imaged on the ChemiDocTM Touch Imaging System (BIO RAD, 1708370). Gel analysis was done using Image Lab (v6.1) to assess band intensity. %AT was determined by subtracting the annotated terminated band from 100%.

## Results

### A bioinformatic pipeline to discover transcriptional fluoride riboswitch variants

To begin this study, we first developed an accessible bioinformatic pipeline to find fluoride riboswitch variants that are predicted to regulate at the transcriptional level. Most bioinformatic analysis performed on riboswitch sequences is done on the highly conserved aptamer domains, which perform a single function of binding a specific ligand. RNA structure databases, such as Rfam [45], only contain information on the aptamer sequence and structure and do not contain information about the downstream EPs, which can vary in function, sequence and structure. To analyze transcriptional EPs of riboswitch variants, we first created a bioinformatic pipeline to collect genomic sequences downstream of the aptamers that could contain EPs. We then computationally predicted whether these EPs could contain an intrinsic terminator.

Our pipeline consisted of several steps (Fig. 1C and “Methods” section): (i) collect aptamer sequences from the fluoride riboswitch Rfam family (RF01734); (ii) extend aptamer sequences to include the downstream EP sequence using aptamer genomic coordinates; and (iii) computationally predict whether the extended EPs could contain intrinsic terminators. Aptamer sequences were acquired from a representative pool of fluoride aptamers from the RNA structure family database, Rfam [45], which results in a collection of 2358 aptamer sequences (Fig. 1C). For each aptamer, we next extended its sequence up to a length that fit within the 300 nt limit for DNA pool synthesis. This was done using the NCBI accession ID and aptamer genomic coordinates within the Rfam FASTA file of aptamer sequences. After extending the sequences, we were left with a collection of 1901 potential riboswitch variants since non-NCBI entries meant there was no genomic context from which to acquire downstream sequence (e.g. URS0000BC4686_32630 from [15]) (Fig. 1C). To check that the extended sequences capture all the downstream EP, we bioinformatically searched for the location of the start codon of the first open reading frame downstream of each aptamer (Supplementary Fig. S5). This analysis showed that 1566/1901 (82%) riboswitch sequences had start codons within the DNA pool synthesis limit, suggesting that our pipeline captures the complete EP for most sequences.

To identify which riboswitch variants potentially regulate gene expression using intrinsic termination, we next analyzed extended EP sequences with the accessible webserver ARNold [46]. The ARNold webserver predicts intrinsic termination through two algorithms: Erpin, which is trained on terminator sequences from *Bacillus subtilis* and *E. coli* [56], and RNAmotif, which is a descriptor with a score cutoff to identify terminator hairpins [57]. The ARNold webserver predicted that 536 (28%) of the fluoride aptamers in the pool were coupled to an EP that specifically folds into an intrinsic terminator (Supplemental Document A). These terminators had a predicted free energy range from −17.1 to −2.5 kcal/mol, with an average of –10.17 kcal/mol (Fig. 1D and Supplemental Table S4), which falls within expected values [58, 59]. Furthermore, the full riboswitch sequences, including aptamer and extended EP, were an average of 98 nts in length, which is within the DNA pool synthesis limit (Supplementary Fig. S6). While ARNold did not identify terminators for the majority of riboswitch variants, it showed potential as an accessible means to identify transcriptional riboswitches. We next sought to alternatively identify transcriptional fluoride riboswitches from all 1901 bioinformatically captured sequences.

### A high-throughput next-generation sequencing pipeline to measure intrinsic termination of 1901 riboswitch variants

To experimentally assess termination of each of the 1901 fluoride riboswitch variants, we converted the *in silico* generated riboswitch sequences into a pool of DNA transcription templates using massively parallel oligonucleotide synthesis [41]. To each riboswitch sequence, we added a 60 nt sequence to the 5′ end that contains the J23119 consensus *E. coli* promoter sequence. We also added a 20 nt 3′ primer binding site for oligo pool amplification. After synthesis, we sequenced the oligo pool to characterize sequence variant coverage, which showed that each member was represented in the pool, with several sequences overrepresented likely due to their shorter length (Supplementary Fig. S7 and Supplemental Document A) [60].

We next amplified the oligo pool into a dsDNA template library and performed an IVT in either 10 mM NaCl or 10 mM NaF. The resulting RNA pool was then processed in a series of preparation steps for NGS (Fig. 2A). Sequencing reads were processed by aligning them to riboswitch variant IDs, mapping the end of the sequencing read to the genomic position, and using this genomic position to classify the read as either terminated or anti-terminated (Supplementary Fig. S8 and the “Methods” section). Two methods were employed to determine sites of termination, either a regular expression search for polyU or the output from BacTermFinder, a convolutional neural network trained on 41 000 bacterial terminators determined from RNA-seq technologies [55] (Supplementary Fig. S9A and the “Methods” section). We found the outcome to be similar between downstream analysis methods (Supplementary Fig. S9B), with BacTermFinder identifying fewer terminators than the polyU regular expression (Supplementary Fig. S9C). A plot of the normalized read counts for each variant across replicates showed strong correlations with *R*^2^ of 0.97 (10 mM NaCl) and 0.99 (10 mM NaF) (Supplementary Fig. S10A and B).

Given recent work highlighting the dynamic relationship between transcription factors and riboswitches [39, 43, 44], we decided to test a previously unexplored transcription elongation factor to identify novel crosstalk between riboswitches and transcription. We supplemented the IVT reaction with 1.2 µM of the highly evolutionarily conserved transcription elongation factor, GreB, which rescues stalled RNAPs that have backtracked by cleaving the 3′ end of the RNA transcript in the RNAP active site [42]. To confirm that the addition of GreB alters our transcription reaction, we performed IVT of a three-way junction dimeric broccoli construct [51], which showed that GreB increases transcriptional output (Supplementary Fig. S11). We next repeated transcription, processing, sequencing, and analysis of the DNA template pool with 1.2 µM GreB. Again, we found replicate experiments with GreB highly reproducible with *R*^2^ of 0.98 (10 mM NaCl) and 0.98 (10 mM NaF) (Supplementary Fig. S10C and D).

To identify intrinsic termination events, we wrote a Python script that classifies reads as “terminated” if they were within the mid-fifteen nucleotides of the top scoring BacTermFinder termination window, and “anti-terminated” if they ended beyond the termination site for that variant (“Methods” section). All reads for a variant are classified as “anti-terminated” if the top score was below 0.47 for a strict threshold as recommended. To reduce noise, the number of “terminated” reads had to be over 5%. The variants classified as “terminated” was consistent between replicates (Supplementary Fig. S12). A confusion matrix comparing the average number of variants with measured termination without GreB (1162 variants after averaging the replicates) versus those predicted to contain terminators by ARNold showed that 71.7% of terminators were shared between the two methods (Fig. 2B). The major failure mode of the prediction was the 24.8% of variants predicted to not terminate while they were measured to undergo some termination (Fig. 2B). We used RNAStructure [61] to assess terminator structures of a random selection of riboswitches that were measured to terminate. Those variants that were successfully predicted by ARNold had a perfect hairpin whereas the unsuccessful predicted variants had a mismatch within the terminator hairpin stem (Supplementary Fig. S13). When ARNold successfully predicted a terminator, we found it matched the output from BacTermFinder (slope = 0.98, *R*^2^ = 0.98) (Supplementary Fig. S14).

For terminating variants, riboswitch lengths were calculated by taking the median read length within the termination window (Supplemental Document A). The terminator and riboswitch statistics from the NGS assay (Supplementary Fig. S15), were similar to the statistics from ARNold (Supplementary Fig. S6). The majority of riboswitches had downstream start codons 28 nts after the termination site (Supplementary Fig. S15C), further supporting that these variants are putative riboswitches. Together, these demonstrate the large range of terminating variants our assay can detect.

As GreB acts on arrested RNAP complexes across bacterial species [42], we sought to assess how transcription termination frequency changed between the without (0 µM) and with (1.2 µM) GreB reactions (Supplemental Document A). We plotted the shift in termination and observe that variants predominately have termination impacted similarly in both salt conditions (QI, less termination with GreB, and QIII, more termination with GreB, in Fig. 2C) or GreB increases termination in the NaCl condition but decreases termination in the NaF condition (QII in Fig. 2C). We also tested for significant changes using Student’s *t*-test (see “Methods” section) and counted which variants had a *P*-value <.05 and greater than 20% change in termination (Fig. 2D). Notably, only 10 variants significantly change >20% in both salt conditions.

Overall, we implemented an NGS pipeline to identify transcriptional termination sites in riboswitch variants that we could then validate for functionality.

### Comparison of high-throughput NGS characterization of riboswitch function with IVT

**Figure 3.**
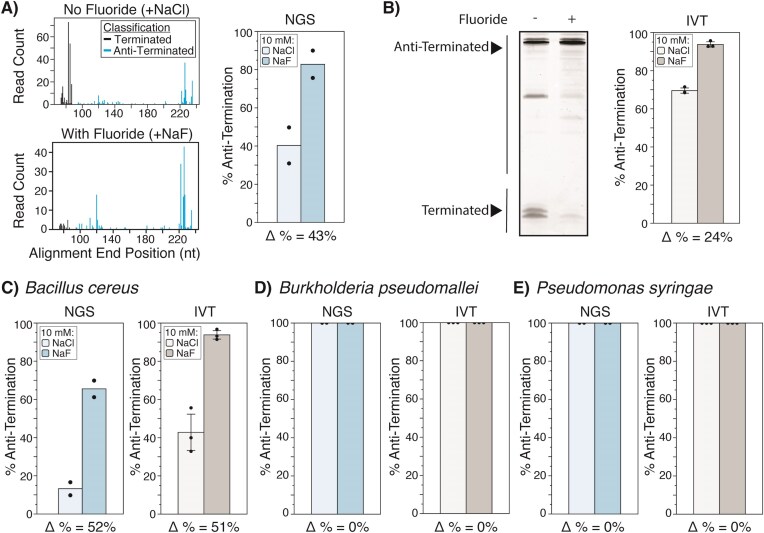
Validation of high-throughput versus IVT characterization of published fluoride riboswitch function. (**A**) NGS analysis of the *Thermotoga petrophila* (accession ID: CP000702.1/1794817-1794880) riboswitch. Plots represent read alignments classified as terminated or anti-terminated in 10 mM NaCl (left, top) or NaF (left, bottom) in the presence of 1.2 µM GreB. The %AT was calculated as 1 − (terminated count)/(anti-terminated count + terminated count) and plotted in a bar graph with two replicates (right). The change (Δ) of percent (%) anti-termination is annotated on the plot. (**B**) IVT quantification (with 1.2 µM GreB) of the *Thermotoga petrophila* variant. The %AT was calculated by quantifying the band intensities of the regions annotated as anti-terminated and terminated (left) using the formula from panel (A) and plotted in a bar graph (right). NGS- and IVT-quantified %AT for (**C**) *Bacillus cereus* (accession ID: CP000227.1/4763720-4763779), a transcriptional control, (**D**) *Burkholderia pseudomallei* (accession ID: BX571966.1/2539005-2538939), a translational control, and (**E**) *Pseudomonas syringae* (accession ID: AE016853.1/5215709-5215637), a translational control. Bars represent average %AT with error bars representing ± standard deviation (*N* = 2 for the high-throughput NGS assay, *N* = 3 for the low-throughput IVT assay). Structures for aptamer and EPs and high-throughput data without GreB are in Supplementary Fig. S16. Annotated gel images: Supplementary Fig. S20.

We next sought to validate our NGS pipeline by comparing the quantification of %AT with and without NaF with that quantified by low-throughput IVT experiments. For a particular riboswitch variant in the NGS assay, we calculated a %AT value by classifying sequence reads as “terminated” or “anti-terminated” based on the read alignment end position and calculated %AT by dividing the number of terminated reads by the sum of all the categorized reads and subtracting from 1 in both NaCl and NaF conditions (Fig. 3A). This NGS quantification was then compared to individual variant IVT experiments by running IVT outputs on a denaturing PAGE gel, classifying bands as “terminated” and “anti-terminated” based on length, and calculating %AT based on band intensities (Fig. 3B). We then calculated a change in %AT (Δ %AT) by subtracting the NaCl condition from the NaF condition.

We used this method to validate the NGS approach using two transcriptional fluoride riboswitch variants [62], *Thermotoga petrophila* (CP000702.1, *T. pe*; Fig. 3A and B, and Supplementary Fig. S16A) and *B. cereus* (CP000227.1, *B. ce*; Fig. 3C and Supplementary Fig. S16B). For *T. pe*, we observed a Δ %AT of 43% in the NGS assay but 24% in the IVT assay driven by a lower %AT value in NaCl condition. The comparison was much closer for *B. ce*, where we observed a Δ %AT of 52% in the NGS assay and a 51% in the IVT assay, although the %AT is higher in the IVT assays. We next validated our approach using two translational fluoride riboswitch variants [63], *Burkholderia pseudomallei* (BX571966.1, *B. ps*; Fig. 3D and Supplementary Fig. S16C) and *P. syringae* (AE016853.1, *P. sy*; Fig. 3E and Supplementary Fig. S16D). As expected for both, we observed a Δ %AT of 0% in both the NGS and IVT assays.

Overall, these results show that the NGS assay accurately classifies riboswitches as using intrinsic termination, though exact values of %AT differ when compared to more traditional IVT assays.

### Discovering highly functional transcriptional riboswitches with NGS

**Figure 4.**
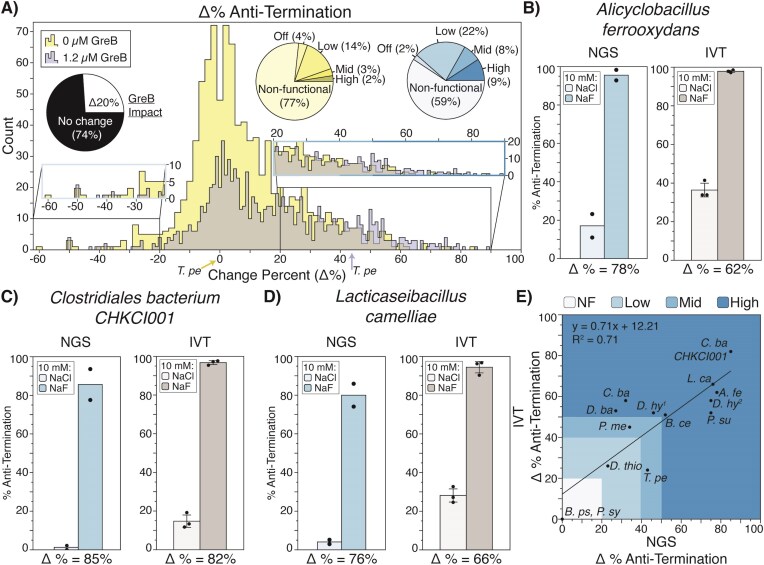
Estimating fluoride riboswitch function from high-throughput sequencing assay. (**A**) Distribution of change (Δ) in the %AT measured by the NGS assay for variants with detected intrinsic termination. Distributions are shown from assays without GreB (yellow), and with 1.2 µM GreB (blue) (overlap = gray). The dynamic range in response to fluoride, Δ%AT (change in percent anti-termination), was determined by averaging the %AT between each condition replicate and then calculating NaF %AT − NaCl %AT. Variants were classified according to calculated Δ %AT ranges: “Off” (−60% to −20%), “non-functional” (−20% to 20%), “low functional” (20%–40%), “mid functional” (40%–50%), and “high functional” (50%–100%). Pie charts show classification percentages for each GreB condition. Arrows labeled “*T. pe*” designate the *Thermotoga petrophila* switching function in 0 µM (blue) and 1.2 µM (yellow) GreB. IVT validation of NGS assay results for *Alicyclobacillus ferrooxydans* [*A. fe*; LJCO01000051.1/58159-58221, (**B**)], *Clostridiales bacterium CHKCI001* [*C. ba CHKCI001*; FCNS01000019.1/7032-7129, (**C**)], and *Lacticaseibacillus camelliae* [*L. ca*, AYZJ01000062.1/6126-6187, (**D**)]. Data shown on the blue bar graphs are from the two biological replicates (*N* = 2) of the high-throughput sequencing assay with 1.2 µM of GreB. Data shown on the gray bar graphs are from three (*N* = 3) replicates of single-round IVT reactions with 1.2 µM GreB. Both assays are done in the presence of either 10 mM NaCl or NaF. Data are presented as %AT with ± standard deviation. Under each graph is the change (Δ) of %AT between the NaF and NaCl conditions for the dataset (Supplemental Document A). Structures for aptamer and EPs and high-throughput data without GreB are in Supplementary Fig. S17. (**E**) Correlation of the average Δ %AT in the NGS (*x-*axis) versus IVT (*y*-axis) measurements. Points are plotted for 10 riboswitches and labeled corresponding to their species (Supplementary Table S6 and Supplementary  Figs S16–18; D. hy^1^ = FWXF01000014.1, D. hy^2^ = FWXF01000003.1). Colors show riboswitch functional classification. The line of best fit is drawn with a slope of 0.71 and a *y*-intercept of 12.21% and an extracted *R*^2^ = 0.71. Graphing calculations done in the DataGraph (v4.5.1) software. Gel images are shown in Supplementary Figs S19–S22.

We next sought to evaluate the function of the identified transcriptional fluoride riboswitches, i.e. the difference of anti-termination (AT) between the NaCl and NaF conditions. For each variant, we calculated the change of %AT (“Δ%AT”) by subtracting the %AT in the NaCl condition from the %AT in the NaF condition. We then used the calculated Δ%AT to classify each variant in both 0 µM and 1.2 µM GreB conditions as “Off” (−60% to −20%), “non-functional” (−20% to 20%), “low functional” (20%–40%), “mid functional” (40%–50%), and “high functional” (50%–100%). Each category has several variants with a majority of riboswitch variants in the “non-functional” category (Fig. 4A), which could be due to incompatibilities with riboswitch folding and the *E. coli* RNAP used in this experiment.

As we observed that GreB impacts transcription termination in this assay (Fig. 2C and D), we next compared how the addition of GreB affects riboswitch function. Interestingly, when GreB was present, 26% of variants had an increase in Δ %AT of at least 20% (Fig. 4A), suggesting GreB is important for this subset of variants. There is no clear sequence motif in this subset indicating why GreB was impactful because of the different outcomes of GreB on termination (Fig. 2C and D).

We next sought to establish how individual variants characterized in the high-throughput NGS assay perform when individually isolated in the low-throughput IVT assay. We selected several riboswitch variants in each functional category, and not currently characterized in the literature to our knowledge (Fig. 4B–D and Supplementary Figs S16–S18), as well as several variants with a greater dynamic range than the current fluoride riboswitch model variant, *B. ce* (Fig. 3C) [4, 37–39, 62, 64]. Correlating the Δ %AT values measured by the NGS and IVT assays (Fig. 4E), shows a strong correlation (*R*^2 ^= 0.71), but with a slope of 0.71, showing that the NGS assay correctly categorizes each variant, but overestimates the quantitative value of Δ %AT compared to IVT. This could be due to a function of the different assay conditions in NGS, with all riboswitches being transcribed simultaneously, or due to steps in the NGS library preparation that could introduce bias, for example by favoring shorter RNA fragments during amplification and flow cell clustering [60, 65].

Ultimately, these results demonstrate that the high-throughput NGS assay can accurately categorize nearly two-thousand riboswitch variants into functional categories in a single experiment, and that it can be used to investigate how transcription conditions (i.e. the addition of GreB) affects the performance of these systems. This leads to massively parallel riboswitch characterization and identification of highly functional switches, such as the *C. bacterium* fluoride riboswitch with a Δ %AT = 82%, which is the highest we have observed.

### Using experimentally guided bioinformatics to develop a covariation motif for a complete transcriptional riboswitch

Covariation has been instrumental to riboswitch discovery [20, 26] as aptamers have highly conserved structures. Covariation models work by taking an input sequence alignment and searching for positions that co-vary (i.e. an A position changing to a G, and a downstream U position changing to a C), indicating that these changes occur during evolution to preserve base pairing [23, 24]. However, covariation analysis has been difficult to apply to riboswitch EPs given their wide sequence and functional variability [20]. To address this limitation, we developed an approach to filter EPs that function as transcription terminators through either computational (Fig. 1) or experimental (Fig. 2) means. We hypothesized that this functional filtering would constrain the EP sequences enough to be able to develop a covariation motif for the entire transcriptional fluoride riboswitch sequence including both the aptamer and terminator.

**Figure 5.**
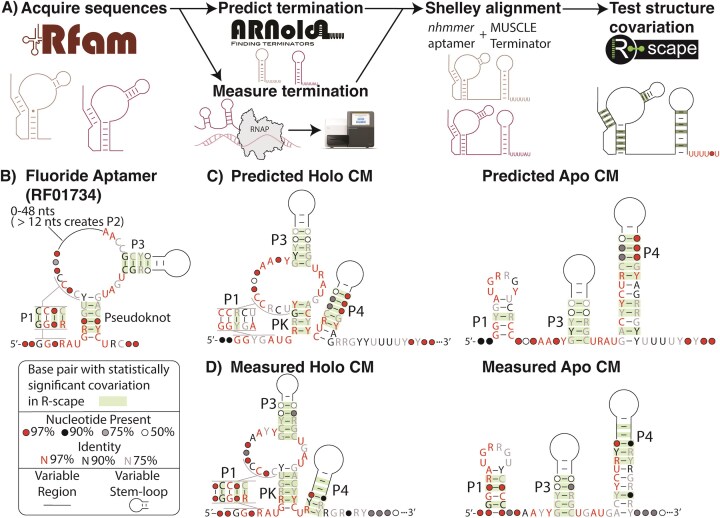
Deriving an experimentally informed covariation model of the fluoride riboswitch aptamer and EP. (**A**) Computational pipeline to establish a covariation model. Fluoride aptamer sequences were acquired from Rfam family RF01734, and extended by ~150 nts (Fig. 1C). EP sequences were filtered to preserve those that contain an intrinsic terminator using either computational prediction by ARNold (Fig. 1), or through experimental validation (Fig. 2). The alignments for the aptamer and the terminator were generated separately and combined then analyzed by R-scape to generate a covariation model. (**B**) Covariation motif of the fluoride aptamer from Rfam (RF01734). Green highlighting on base pairs denotes evolutionary significance in base-pair covariation, and nucleotides are denoted as circle or specific letters with colors to signify conservation: red = >97%, black = 90%–97%, gray = 75%–90%, and white = 50%–75%; R = purines: A or G. Y = pyrimidines: U or C; P# = pairing element. (**C**) Covariation motif of transcriptional fluoride riboswitches predicted to undergo intrinsic termination in the Holo (ligand-bound, left) or Apo (ligand-free, right). The two alternative stems are depicted as PK (pseudoknot) and P4 (terminating stem). The structures have been derived from Supplementary Fig. S23A showing the full R-scape output. (**D**) Holo (ligand-bound, left) or Apo (ligand-free, right) configurations for variants measured to terminate with the NGS assay. The two alternative stems are depicted as PK (pseudoknot) and P4 (terminating stem). The structures have been derived from Supplementary Fig. S23C showing the full R-scape output. The dashed line in panels (C) and (D) highlights the EP sequence known to be directly involved in the riboswitch structural switching mechanism. Covariation motifs adapted from raw output in Supplementary Fig. S23.

From the functionally filtered sequence, we perform two alignments: the aptamer portion using the nhmmer command in R-scape [24, 47] and the sequence after the aptamer to the termination site using MUSCLE [48]. We then devised the Shelley method to produce “Frankenstein” alignments for the aptamer and terminator to create a combined aptamer-terminator alignment. Covariation was assessed using R-scape [24] (Fig. 5A). The holo structure, when ligand is bound, was the automatic output of CaCoFold; whereas the apo structure, when ligand is unbound, was drawn with the alternative covarying pattern (Supplementary Fig. S23).

The covariation motif of the fluoride aptamer previously identified (RF01734) [45] contains three helixes covering at least three significant covarying base-paired regions: P1, pseudoknot, P3 (Fig. 5B). In comparison, the covariation model using the transcriptional riboswitch variants predicted by ARNold largely recreated the aptamer model by identifying P1, the pseudoknot, and P3 (Fig. 5C). There are slight changes in the length of the base-paired (P) regions and more overall sequence conservation within the aptamer (Fig. 5C). The EP model did result in an intrinsic terminator motif that in the apo state includes a hairpin and a downstream polyU motif (Fig. 5C). Notably, purines are preferred on the 3′ end of the terminator, adjacent to the polyU, as similarly observed for the purine [34] and SAM [35] riboswitch covariation motifs but not in general terminators [66]. We also identified sequence covariation along the terminator involving nucleotides that comprise the pseudoknot in the holo state (Fig. 5C), in accordance with several studies on the riboswitch structural rearrangement mechanism [4, 38, 39, 62]. A model utilizing sequences measured through NGS to be transcriptional riboswitch variants resulted in a similar model, but with overall slightly less covariation in the terminator (Fig. 5D), potentially due to the increased number of sequences in the model.

Overall, our bioinformatic approach demonstrates a high-throughput and accessible construction method of a covariation model for a complete riboswitch by filtering for a specific EP function. The fact that this model contains elements consistent with the known switching mechanism of the fluoride riboswitch suggests this could be a powerful approach to uncovering the mechanism of other riboswitch classes.

### Covariation models of full riboswitch aptamer and expression platforms for other classes reveal common riboswitch aptamer and downstream expression platform motifs

The similar riboswitch covariation motifs we observed between both terminator filtering methods supports the use of ARNold for rapid and high-throughput generation of complete covariation motif for other riboswitch classes. We therefore applied our approach to generate the complete motifs for several additional riboswitch classes: purine [34] and SAM [35] for their prior covariation characterization; ZTP, which has been extensively studied in a cotranscriptional context [6, 67]; Lysine, which regulates transcription termination [68–70], translational sequestering, and RNase E cleavage [71]; and TPP classes, which is the most common riboswitch and occurs throughout all three kingdoms of life [20].

To expand our analysis beyond transcriptional riboswitches, we further refined the method by calibrating the ARNold-generated covariation motif of the fluoride riboswitch, consisting of the aptamer and the downstream terminator hairpin, to increase sensitivity and speed in motif searching through sequences by determining the expected value scores and generating filter score cutoffs (cmcalibrate, see “Methods” section). We then searched all the extended sequences in the riboswitch class for the aptamer + hairpin motif (Supplementary Fig. S24A). For the fluoride riboswitch, searching all variants did not produce novel information from the previous results, although the polyU track was reduced to a string of five pyrimidines (Fig. 5C and D, and Supplementary Fig. S24B) suggesting that this covariation motif analysis is able to fit a more diverse set of sequences. We interpreted this as the motif capturing all instances of aptamer + hairpin that covers transcriptional and translational EPs.

We next performed the covariation motif analysis for the purine [34] and SAM [35] riboswitches. For the purine riboswitch, we initially used 101 sequences identified by ARNold to generate a predictive model and then used this model to search all 2611 sequences from Rfam (RF00167). The resulting model recreated the aptamer and revealed a downstream hairpin with 19 out of 21 base pairs covarying (Supplementary Fig. S25A). Notably, the previously published purine covariation motif generated through manually curating an alignment of likely transcriptional variants has higher sequence specificity in the EP, likely due to building a model with 37 variants compared to the 2616 variants in our model [34]. For the SAM riboswitch, we created an initial covariation model with 132 sequences identified from ARNold and then searched the 6114 pulled from Rfam (RF00162) (Supplementary Fig. S25B). Compared to the previously published SAM riboswitch motif [35], we observed the stretch of pyrimidine-purine base pairing pattern in the terminator ahead the polyU but did not observe significant covariation between the aptamer and downstream EP. These results demonstrate our method’s high tolerance for sequence diversity when detecting EPs.

**Figure 6.**
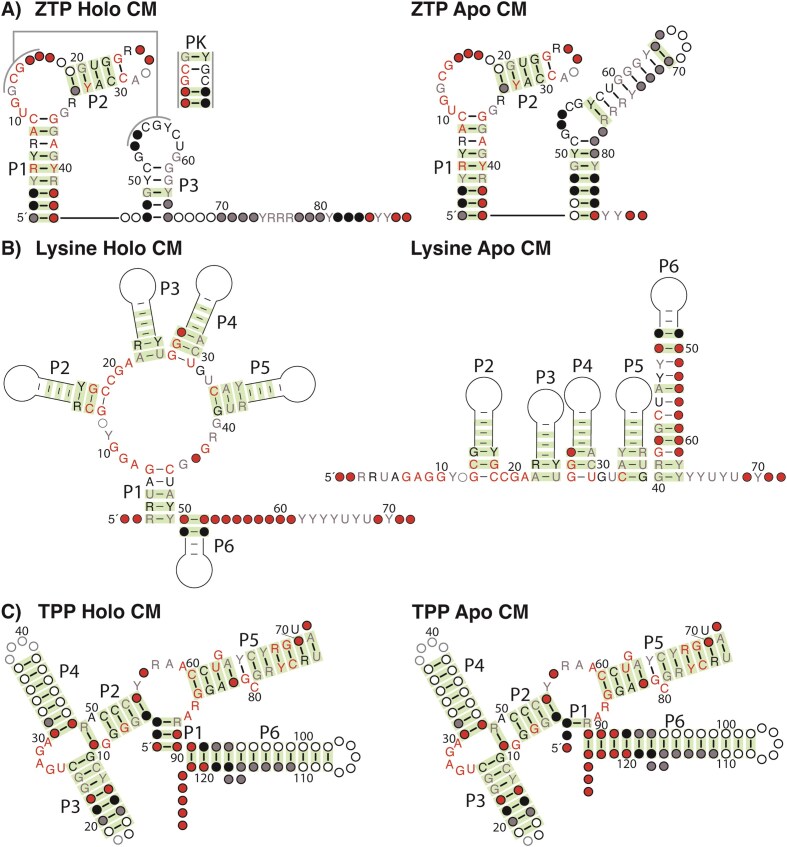
Covariation models of aptamer and EP sequences for ZTP, lysine, and TPP transcriptional riboswitches. Covariation models were generated as in Fig. 5 using the ARNold computational prediction to filter for transcriptional sequence variants. The models were then calibrated and searched for through all downloaded sequences of each riboswitch class (Supplementary Fig. S24). (**A**) The ZTP (RF01750) riboswitch in the holo (ligand-bound, left) or apo (ligand-free, right) state. (**B**) The Lysine (RF00168) riboswitch in the holo (ligand-bound, left) or apo (ligand-free, right) state. (**C**) The TPP (RF00059) riboswitch in the holo (ligand-bound, left) or apo (ligand-free, right) state. Green highlighting on base pairs denotes evolutionarily significance in base-pair covariation, and nucleotides are denoted as circle or specific letters with colors to signify conservation: red = >97%, black = 90%–97%, gray = 75%–90%, and white = 50%–75%; R = purines: A or G. Y = pyrimidines: U or C; P1 = pairing element. The initial R-scape/CaCoFold outputs from the ARNold sequences are in Supplemental Fig. S26. The raw output from CaCoFold is in Supplemental Document C.

We next generated novel riboswitch covariation motifs for additional classes: ZTP riboswitch (Fig. 6A), lysine riboswitch (Fig. 6B), and TPP riboswitch (Fig. 6C). For the ZTP riboswitch, we created an initial covariation motif from 187 sequences identified from ARNold (Supplementary Fig. S26A) and then used it to search the 3458 sequences pulled from Rfam (RF01750) (Fig. 6A). The resulting model showed a rearrangement of the aptamer by the EP, which is supported from previous structural studies [6, 67]. For the lysine riboswitch, the covariation motif was generated from 40 sequences identified from ARNold (Supplementary Fig. S26B) and then used to search the 3458 sequences pulled from Rfam (RF00168) (Fig. 6B). Interestingly, the aptamer–EP covariation overlap was found to include more of the aptamer than present within the *B. subtilis* lysC transcriptional model system [68–70] but matches the *E. coli* lysC translational variant [71] (Supplementary Fig. S27), potentially providing insights into the more common regulatory mechanism. Thirdly, we generated a motif for the highly occurring TPP riboswitch, which similar to ZTP has been used to characterize RNA structural rearrangements outside a cotranscriptional regime context [5]. We created a covariation motif of the TPP riboswitch from 48 sequences identified from ARNold (Supplementary Fig. S26C) and then searched the 16 107 sequences pulled from Rfam (RF00059) (Fig. 6C). The resulting model had only two nucleotides shared between the aptamer and EP (Fig. 6C), which supports the previous rearrangement mechanism [5].

We then generated the covariation motif for the glmS riboswitch (Supplementary Fig. S28), which uniquely regulates gene expression through transcript cleavage rather than an EP [72]. We sought to observe whether there may be glmS riboswitch variants with an EP. While ARNold does identify terminators after the glmS aptamer, there is no covarying overlap between the aptamer or EP (Supplementary Fig. S28), suggesting that an EP method of gene regulation is not commonly used by the glmS aptamer.

Ultimately, this demonstrates that our method can be used to generate covariation models of riboswitches that include both aptamer and EP, and that these models can contain mechanistic insights into how riboswitches structurally rearrange between the aptamer and EP.

## Discussion

In this work, we developed high-throughput methods to generate covariation models of complete riboswitches to investigate the evolutionary conservation of structural switching mechanisms. The core method consisted of a bioinformatic pipeline to acquire full riboswitch sequences predicted to undergo transcription termination (Fig. 1); high-throughput characterization of riboswitch function using NGS readout (Fig. 2); and use of identified transcriptional variants to build covariation models of riboswitch sequences (Fig. 5). Application of this NGS pipeline to the fluoride riboswitch class identified that 323/1901 (17%) of the fluoride riboswitch variants tested were functional in our transcriptional assay. This shows that there are large pools of candidate riboswitch sequences that can be used to study mechanisms beyond the handful of examples typically studied [4, 38, 39, 62]. Furthermore, the covariation model of the complete fluoride riboswitch showed conservation patterns in the EP that directly overlap with those of the aptamer, consistent with the previously uncovered mechanism of the fluoride riboswitch entailing switching between mutually exclusive aptamer/EP folds [62].

One limitation of our approach is that we utilized IVT with the *E. coli* RNAP. As polymerases from other species can respond differently to transcriptional pause signals [73], and have different requirements for intrinsic transcriptional termination [74, 75], the measured function for variants in our assay would likely change if different transcription conditions, or even cellular assays, were used. To understand this limitation, we generated a phylogenetic tree and annotated the termination results (Supplementary Fig. S29), which shows both termination methods have wide phylogenetic coverage. Interestingly, the Actinomycetota phylum in the Bacillati kingdom has nearly no transcriptional variants detected whereas the other Bacillati phylum subclasses are mostly transcriptional (Supplementary Fig. S29I). Additionally, the native transcript start site for most of the variants analyzed is unknown and the 5′ sequence ahead of riboswitches can impact their function [31, 32, 76]. Thus, variant functionality may change with differences in the pre-aptamer leader sequence. Ultimately, our method is specifically suited to identifying functional riboswitch variants in an *in vitro* context rather than predicting *in cellulo* function.

We also found that riboswitch function measured by NGS was in-line with manual IVT validation but not in complete agreement. We hypothesize that this is due to biases in NGS library prep that allow shorter sequence products, i.e. terminated products, to be amplified and bind to the flow cell overcompensating the pool (Supplementary Fig. S7 and  Supplemental Document A) [60, 65]. This may explain the discrepancy in dynamic range between the high- and low-throughput comparisons (Fig. 4E). Recently an approach called TECDisplay was developed [77] that uses an alternative approach to address this bias.

One benefit of our approach is that it can help uncover patterns that deviate from the established understanding of riboswitch mechanisms. During transcription, nascent RNAs can undergo dynamic structure rearrangements, which riboswitches have been an ideal model system to study. For the fluoride riboswitch, this dynamic rearrangement occurred through strand exchange of the six nucleotides in the pseudoknotted aptamer into the base of the terminator for the *B. ce* and *T. pe* variants [4, 62, 78]. This mechanism is supported through covariation analysis (Fig. 5C and D). However, our high-throughput analysis also revealed several functional variants of the fluoride riboswitch that extend through the aptamer auxiliary helix, P3 (Supplementary Figs S17A and B, and S18D, E, and G), rather than just the aptamer pseudoknot as observed for *B.ce* and *T. pe*. Such evolution context and exception may be useful in understanding how folding pathways change when an aptamer switches between controlling transcription versus translation [79, 80].

Our high-throughput approach also allowed us to investigate the effect of transcription factors such as GreB on the function of many riboswitch variants all at once. There is a growing body of work studying the dynamic relationship between riboswitches and transcription factors [39, 43, 44] such as GreB, which rescues backtracked *E. coli* RNAP through cleaving the 3′ end of the RNA [42]. GreB has not priorly been studied in riboswitch functionality even though it is an evolutionarily conserved transcription factor [42]. In our assay, we found that the presence of GreB can have selected impact on a subset of fluoride riboswitch sequences. Notably, GreB engages during class II pauses [81], which differs for the class I pause typically observed for intrinsic termination [29], suggesting that our method may reveal novel principles of molecular biology that are unseen at the single-model case study. Toward the single-model case study, the nucleotide–amino acid interactions between a riboswitch and RNAP exit channel was mapped [82], but notably the nucleotides that interact with the RNAP amino acids are not in highly conserved positions of the aptamer. This highlights the sequence-specific, rather than generalizable, ways nascent transcripts fold and influence transcription progression.

The ease of the computational approach allowed us to apply the method to other riboswitch classes. In total, we generated 6 of the current 51 riboswitches on Rfam. Our full covariation motifs validated similar riboswitch mechanisms previously characterized [5, 6, 35, 67, 71]. Thus, such an approach could potentially be used to discover mechanisms for other riboswitches.

Central to this work was the employment of MPOS. MPOS was previously used to test the structural tolerance of the Twister ribozyme and covariation model searching was employed to identify an active ribozyme in mammals [83]. While we have not similarly searched through other genomes for the fluoride riboswitch, we do note that there are fluoride aptamer sequences deposited on Rfam from *Drosophila ficusphila* (KB456680.1), *Spinacia oleracea* (AYZV02264506.1), wheat (*Triticum Urartu*, AOTI010596493.1), and rice (*Oryza sativa Indica Group*, CH399130.1) (Supplementary Fig. S29). Our NGS assay shows functional variants from Spinacia and wheat, however validation in the organism systems is needed given how transcription machinery changes throughout evolution [73, 84]. Furthermore, this MPOS and NGS assay may accelerate addressing the orphan riboswitch problem [20] by screening a large number of riboswitches during candidate ligand searches.

Ultimately, this work progresses covariation analysis for rapid detection of dynamic RNA confirmation states. These pipelines will aid our search for new riboswitch mechanisms, studies for antibiotic targeting, and developing riboswitches for novel biotechnologies.

## Supplementary Material

gkag542_Supplemental_Files

## Data Availability

The scripts to run the analysis are available at GitHub (github.com/LucksLab/Hertz_HighTroughput_Riboswitch_Discovery_2025) and Zenodo at DOI 10.5281/zenodo.20058763 (https://zenodo.org/records/20058763). Code was written with the assistance of ChatGTP (v.3 and v.4) and manually validated and edited. Accession numbers for all sequences are in Supplemental Document A. The NGS data are deposited on the Sequence Read Archive managed by the NCBI under BioProject: PRJNA1359411.
