# Supplementary material for: High-throughput functional profiling and evolutionary covariation analysis of entire riboswitch sequences
Source: Nucleic Acids Res. 2026 Jun 17;54(11):gkag542. doi: 10.1093/nar/gkag542 (PMC13273304; doi:10.1093/nar/gkag542)
Supplement: gkag542_Supplemental_Files [file gkag542_supplemental_files.zip › Hertz_Supplementary_Information_Key.docx]

Supplementary Information – Supplementary Figures and Tables.

Supplementary_Document_A.xlsx – Output from bioinformatics, python read analysis, read calculations, and gel quantification.

Supplemental_Document_B.xlsx – Output for the in vitro fluorescence assay.

Supplemental_Document_C.pdf – Unedited output from R2R with Rscape.

Supplemental_Document_D.pdf – Unedited phylogenetic trees.
