# Supplementary material for: High-throughput functional profiling and evolutionary covariation analysis of entire riboswitch sequences
Source: Nucleic Acids Res. 2026 Jun 17;54(11):gkag542. doi: 10.1093/nar/gkag542 (PMC13273304; doi:10.1093/nar/gkag542)

RF00167_PurineRiboswitch.arnold.cacofold

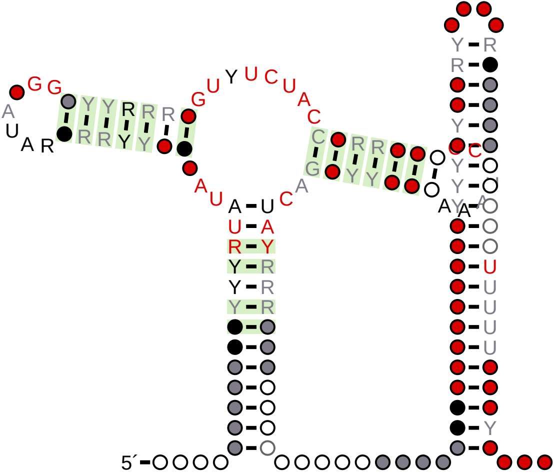

RF00167_PurineRiboswitch.all.cacofold

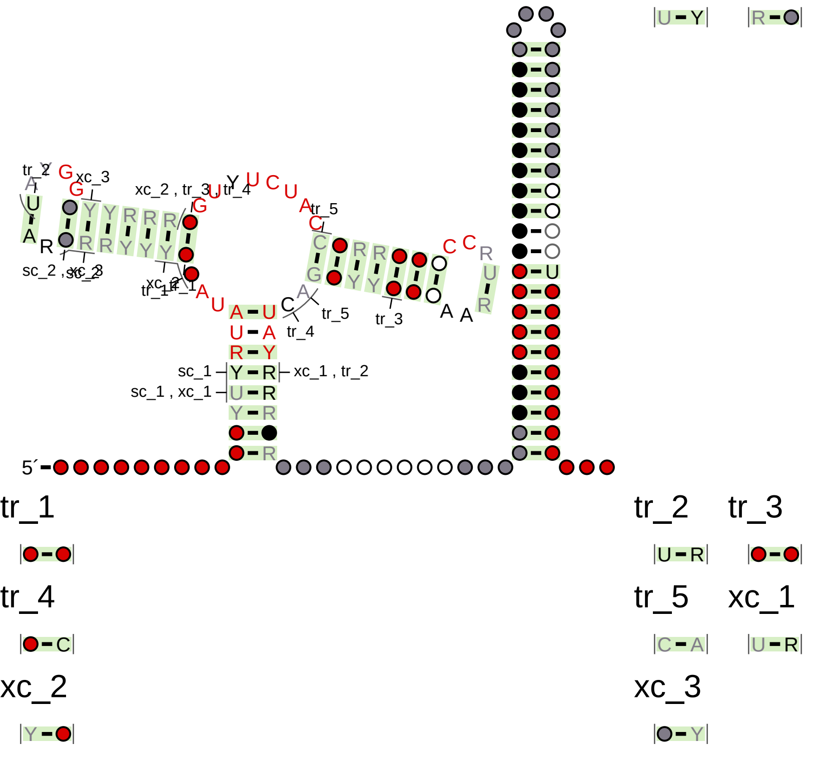

RF00162_SAMRiboswitch.arnold_TruncatedRemoved_1.cacofold

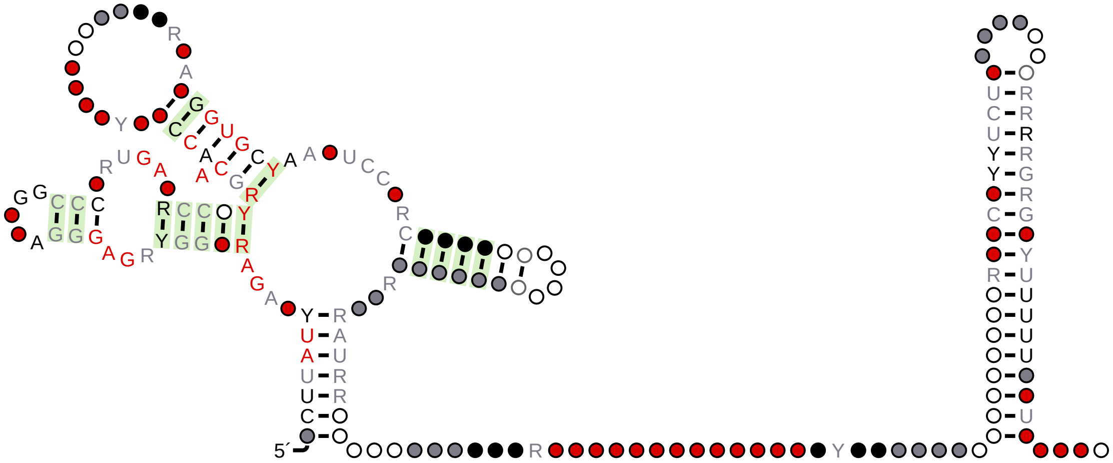

RF00162_SAMRiboswitch.all.cacofold

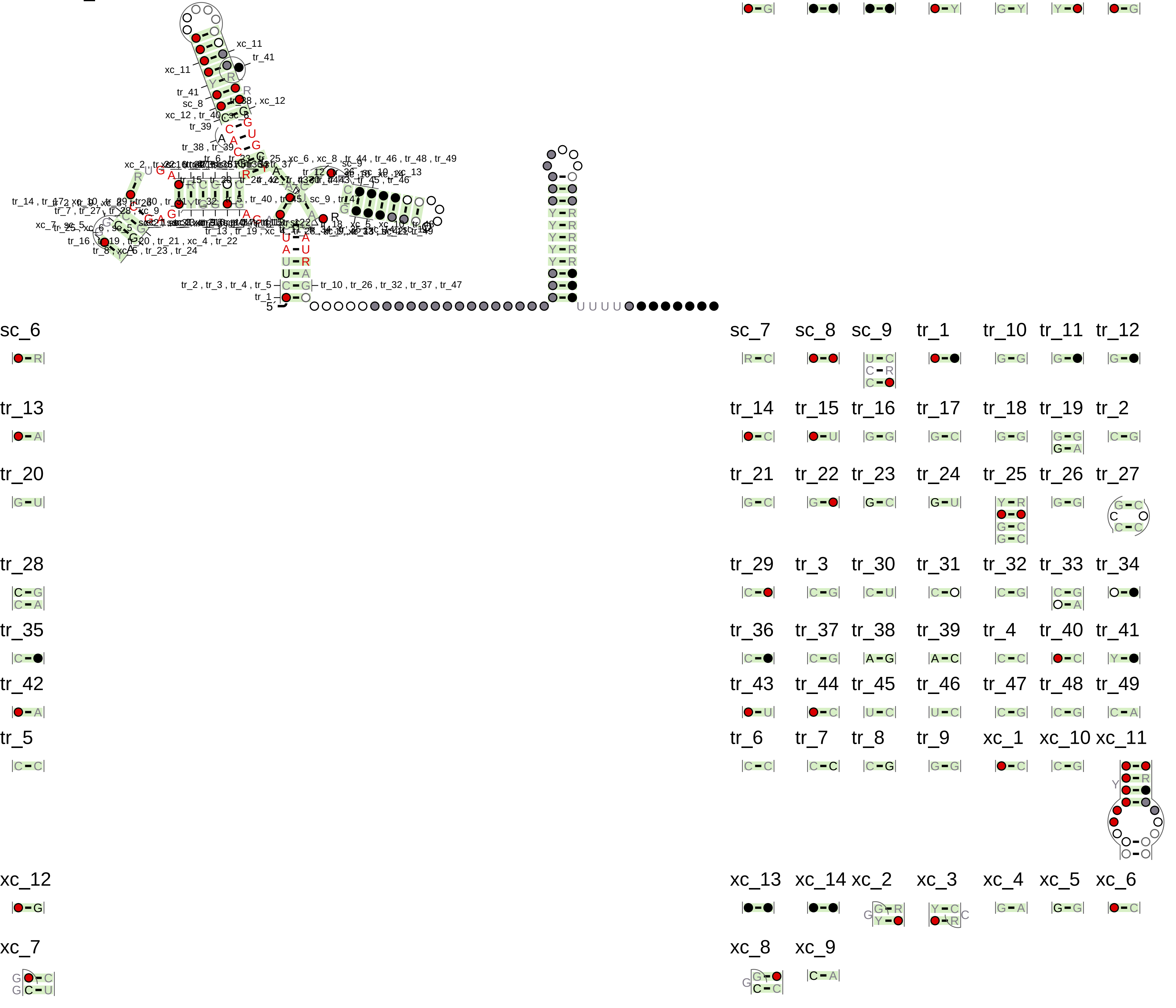

RF01750_ZTPRiboswitch.arnold.cacofold

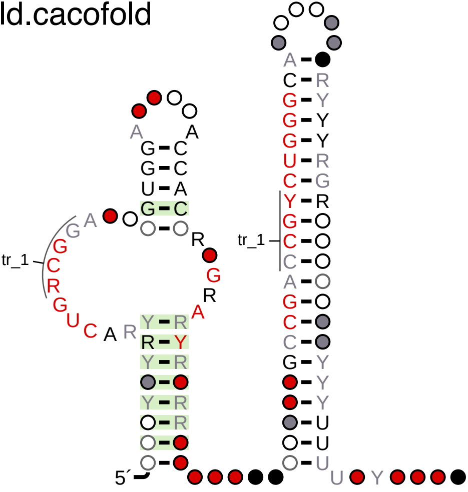

tr_1

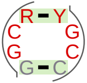

RF01750_ZTPRiboswitch.all.cacofold

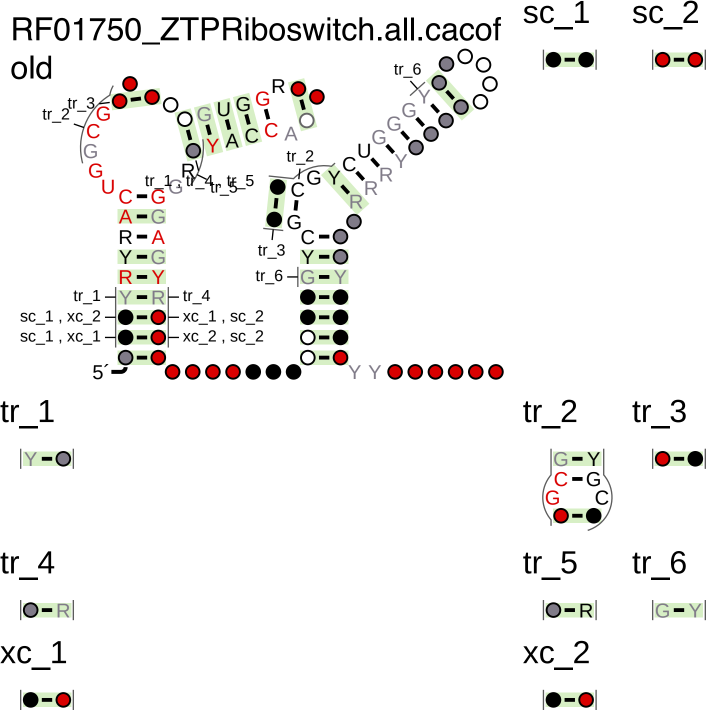

RF00168_LysineRiboswitch.arnold.cacofold

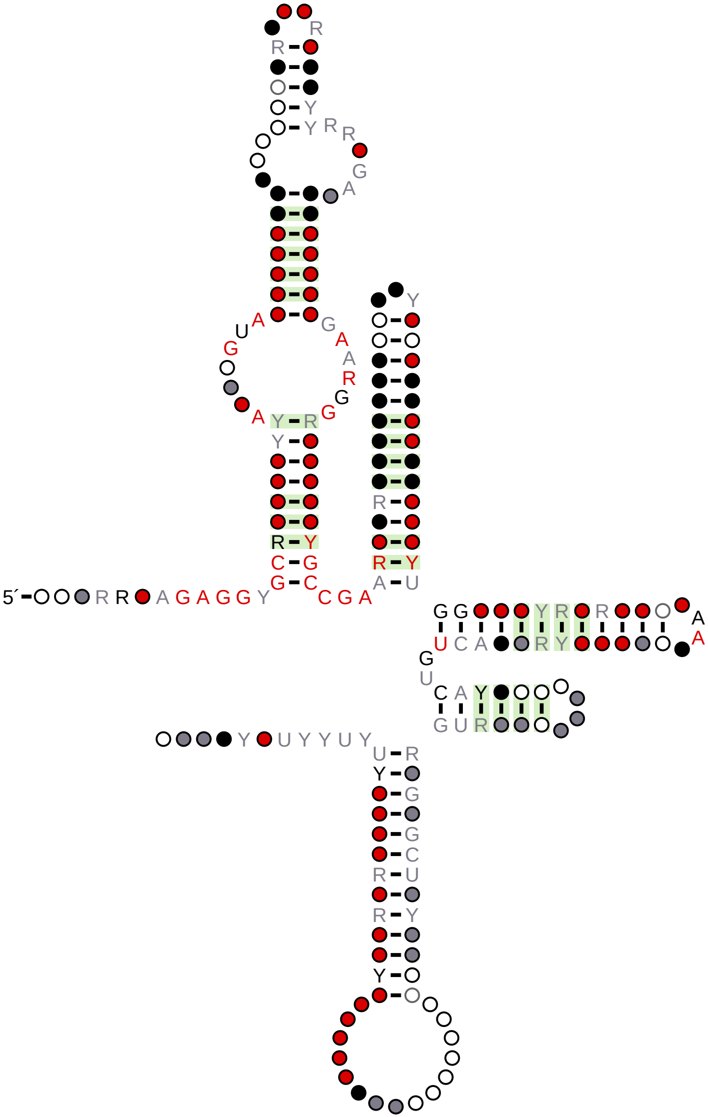

RF00168_LysineRiboswitch.all.cacofold

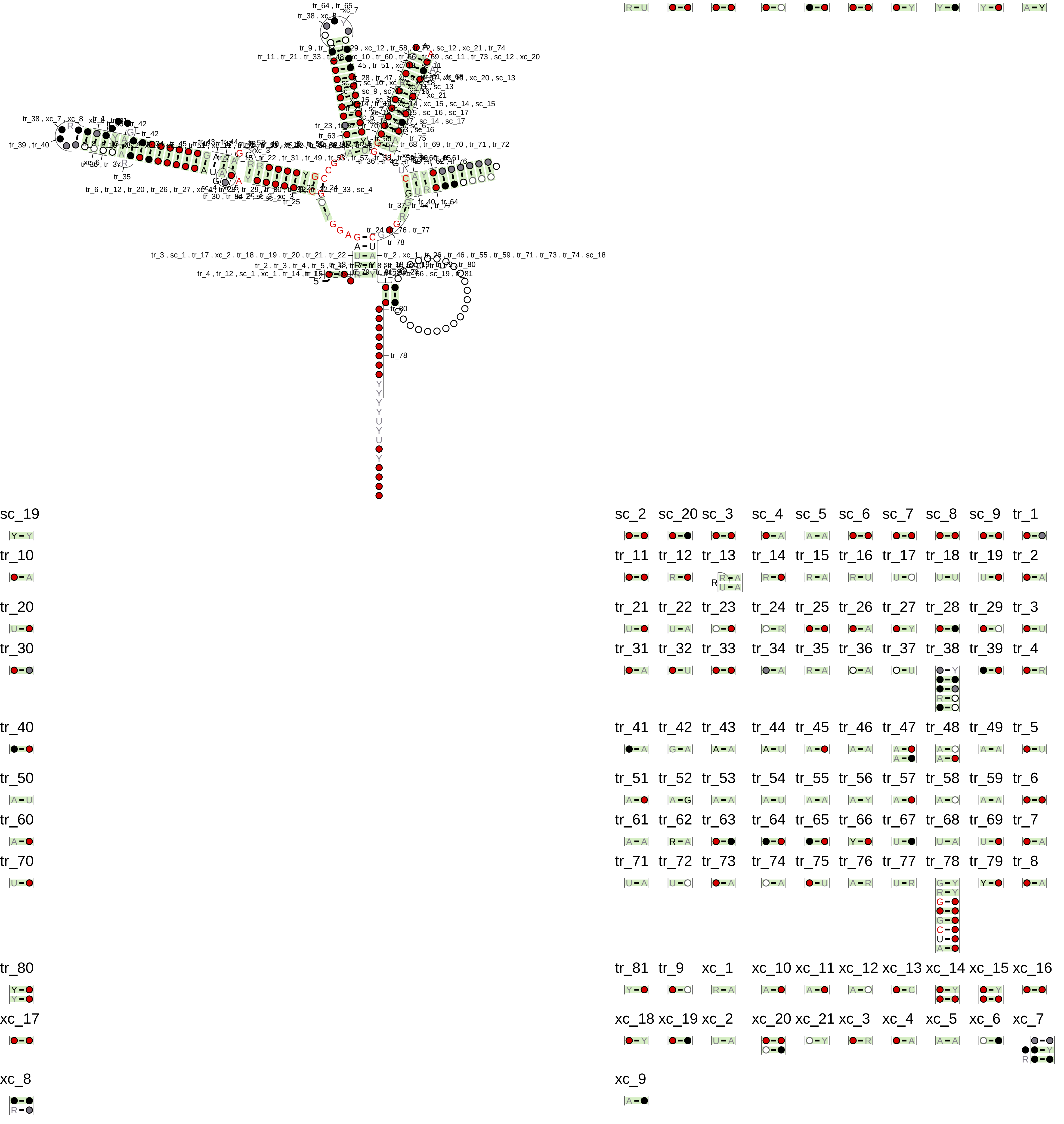

RF00059_TPPRiboswitch.arnold
.cacofold

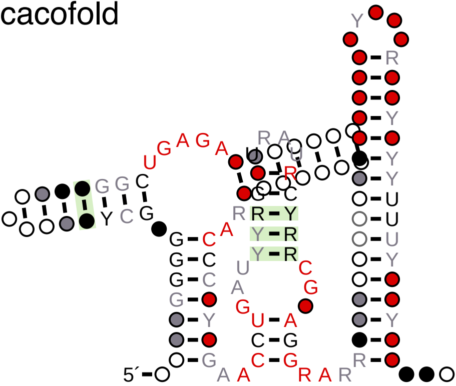

RF00059_TPPRiboswitch.all.cacofold

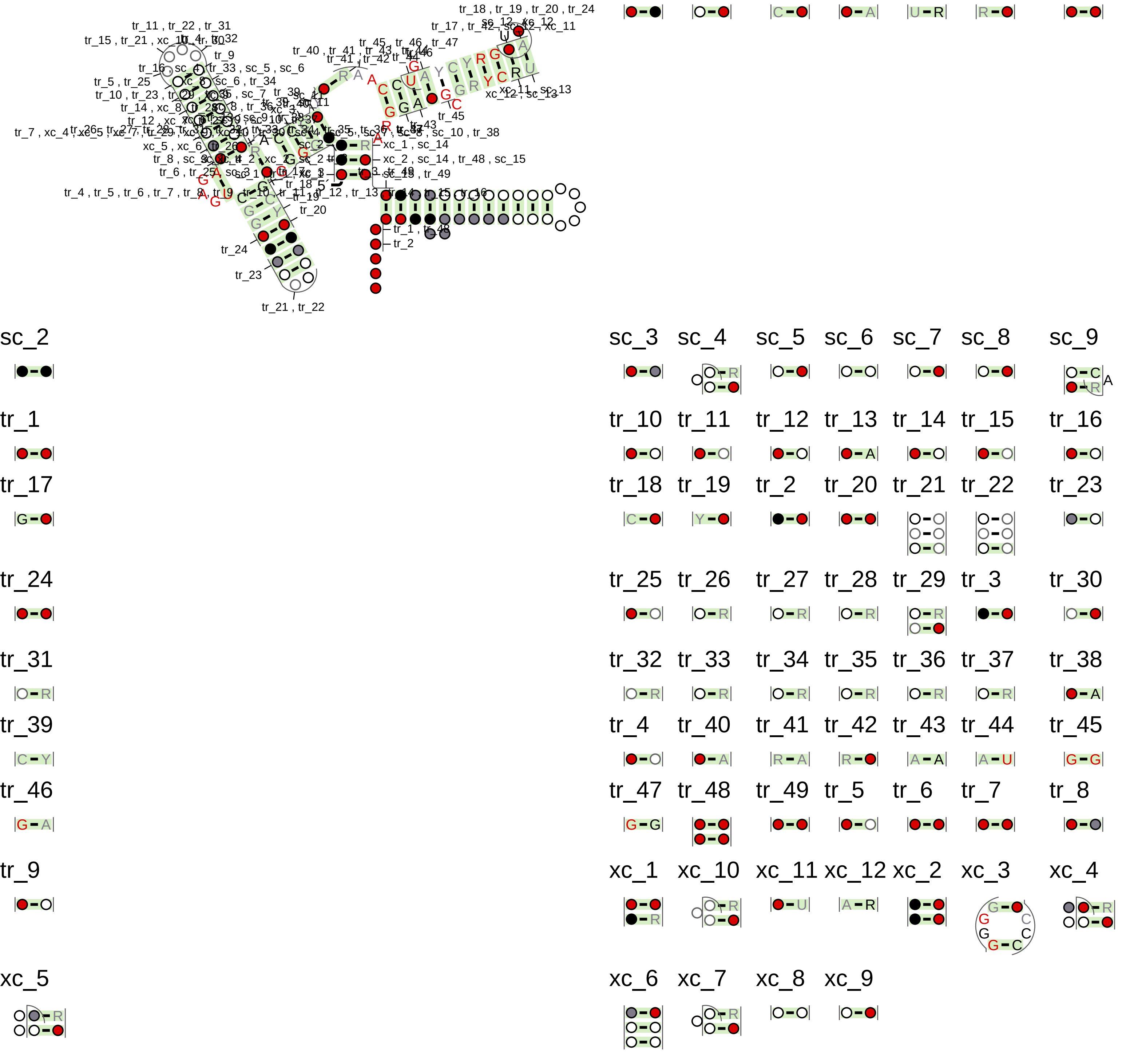

RF00168_glmSRiboswitch.arnold.cacofold

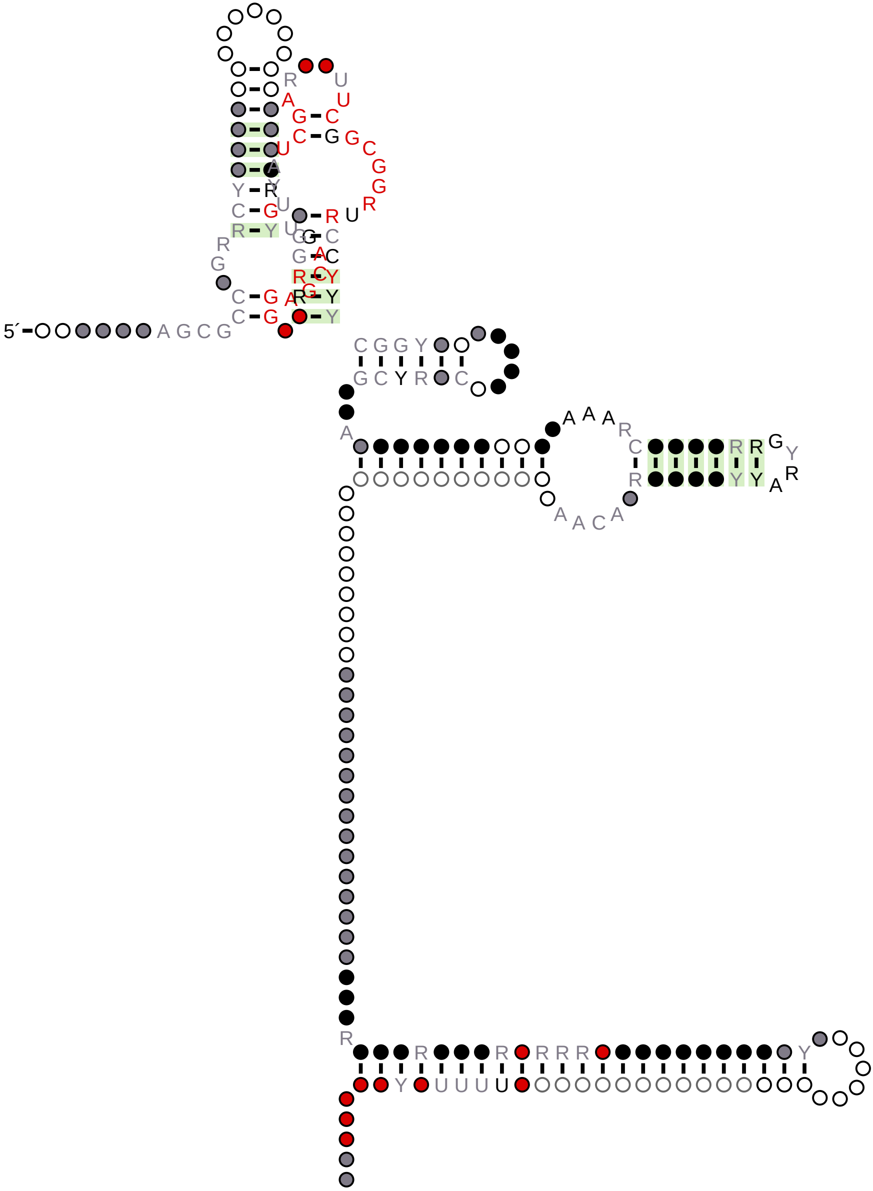

RF00168_glmSRiboswitch.all.cacofold

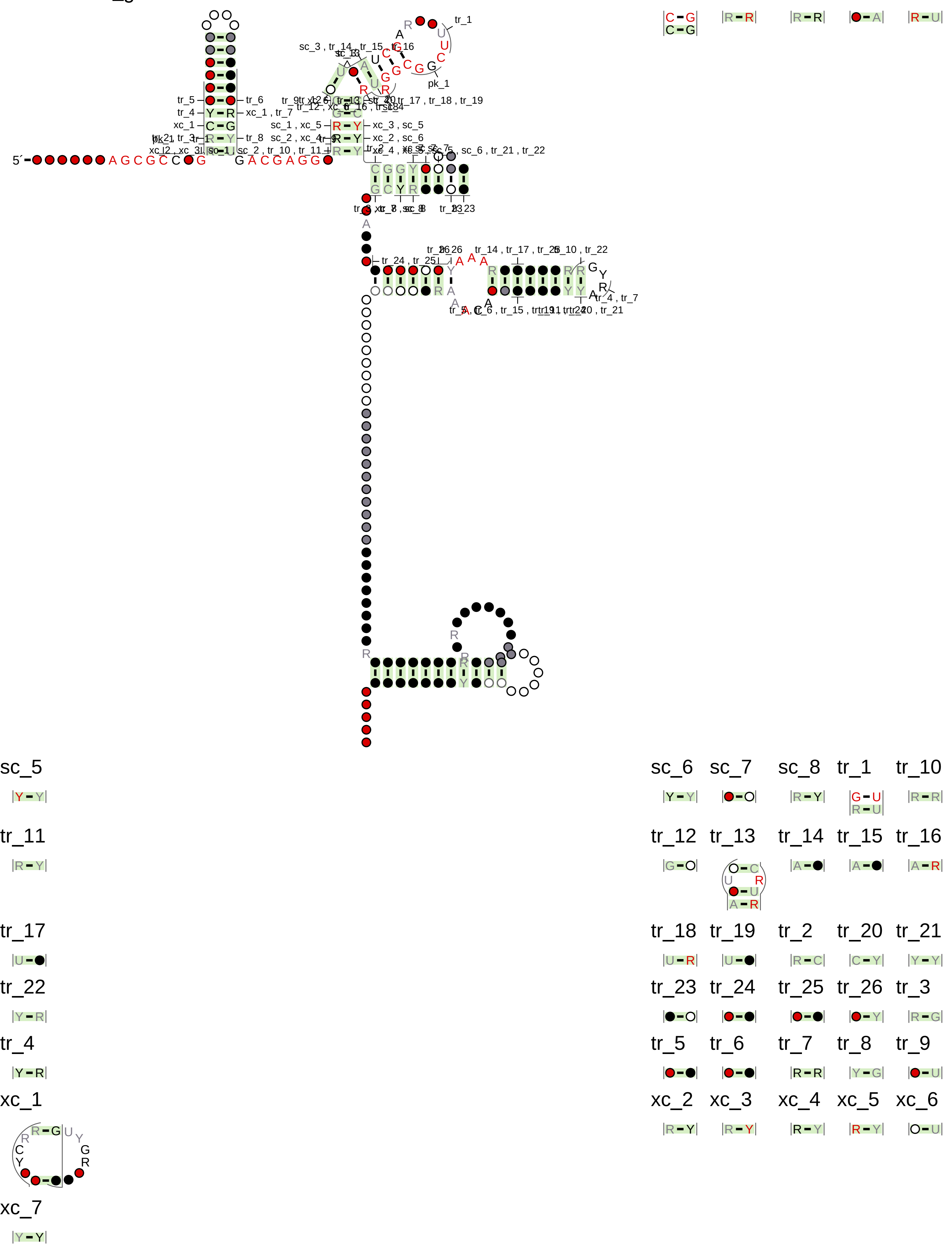

Supplement: gkag542_Supplemental_Files [file gkag542_supplemental_files.zip › Supplemental_Document_C.pdf]
